# Extracellular vesicle-derived silk fibroin nanoparticles loaded with MFGE8 accelerate skin ulcer healing by targeting the vascular endothelial cells

**DOI:** 10.1186/s12951-023-02185-7

**Published:** 2023-11-29

**Authors:** Liwen Luo, Hongyu Zhang, Shiyu Zhang, Chengqin Luo, Xuewei Kan, Jun Lv, Ping Zhao, Zhiqiang Tian, Changqing Li

**Affiliations:** 1 Department of Orthopaedics, Xinqiao Hospital, Army Medical University (Third Military Medical University), 83, Xinqiao St, Shapingba District, Chongqing, 400037 China; 2 Department of Emergency, Second Affiliated Hospital of Chongqing Medical University, Chongqing, China; 3 Institute of Immunology, PLA, Army Medical University (Third Military Medical University), 30 Gaotanyan St, Shapingba District, Chongqing, 400038 China; 4 State Key Laboratory of Silkworm Genome Biology, Biological Science Research Center, Southwest University, 2, Tiansheng Road, Beibei District, Chongqing, 400715 China; 5 Department of Dermatology, First Affiliated Hospital of USTC, Division of Life Sciences and Medicine, University of Science and Technology of China, Hefei, Anhui China; 6 Department of Pharmacy, Southwest Hospital, Army Medical University (Third Military Medical University), Chongqing, 400038 China

**Keywords:** Extracellular vesicles, MFGE8, Pressure ulcers, Silk fibroin nanoparticles, Vascular endothelial cells

## Abstract

**Background:**

Reduced supplies of oxygen and nutrients caused by vascular injury lead to difficult-to-heal pressure ulcers (PU) in clinical practice. Rapid vascular repair in the skin wound is the key to the resolution of this challenge, but clinical measures are still limited. We described the beneficial effects of extracellular vesicle-derived silk fibroin nanoparticles (NPs) loaded with milk fat globule EGF factor 8 (MFGE8) on accelerating skin blood vessel and PU healing by targeting CD13 in the vascular endothelial cells (VECs).

**Methods:**

CD13, the specific targeting protein of NGR, and MFGE8, an inhibitor of ferroptosis, were detected in VECs and PU tissues. Then, NPs were synthesized via silk fibroin, and MFGE8-coated NPs (NPs@MFGE8) were assembled via loading purified protein MFGE8 produced by Chinese hamster ovary cells. Lentivirus was used to over-express MFGE8 in VECs and obtained MFGE8-engineered extracellular vesicles (EVs-MFGE8) secreted by these VECs. The inhibitory effect of EVs-MFGE8 or NPs@MFGE8 on ferroptosis was detected in vitro. The NGR peptide cross-linked with NPs@MFGE8 was assembled into NGR-NPs@MFGE8. Collagen and silk fibroin were used to synthesize the silk fibroin/collagen hydrogel. After being loaded with NGR-NPs@MFGE8, silk fibroin/collagen hydrogel sustained-release carrier was synthesized to investigate the repair effect on PU in vivo.

**Results:**

MFGE8 was decreased, and CD13 was increased in PU tissues. Similar to the effect of EVs-MFGE8 on inhibiting ferroptosis, NPs@MFGE8 could inhibit the mitochondrial autophagy-induced ferroptosis of VECs. Compared with the hydrogels loaded with NPs or NPs@MFGE8, the hydrogels loaded with NGR-NPs@MFGE8 consistently released NGR-NPs@MFGE8 targeting CD13 in VECs, thereby inhibiting mitochondrial autophagy and ferroptosis caused by hypoxia and accelerating wound healing effectively in rats.

**Conclusions:**

The silk fibroin/collagen hydrogel sustained-release carrier loaded with NGR-NPs@MFGE8 was of great significance to use as a wound dressing to inhibit the ferroptosis of VECs by targeting CD13 in PU tissues, preventing PU formation and promoting wound healing.

**Graphical Abstract:**

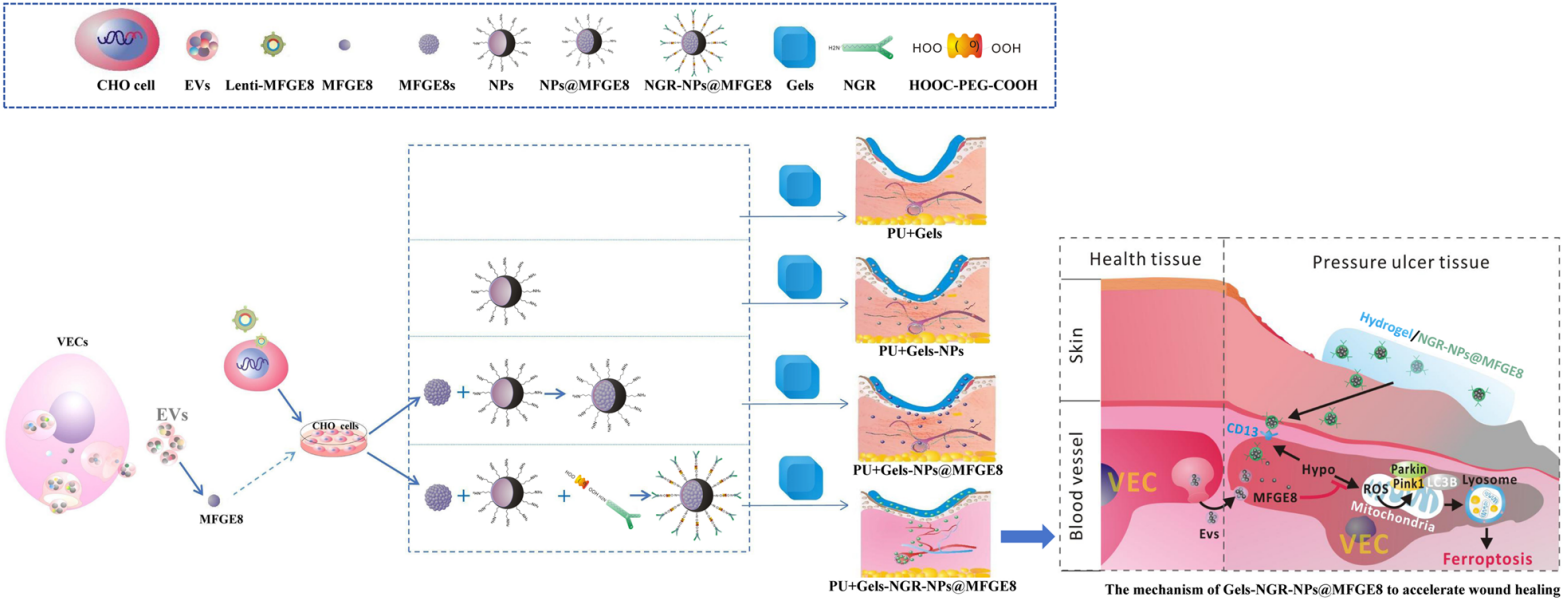

**Supplementary Information:**

The online version contains supplementary material available at 10.1186/s12951-023-02185-7.

## Introduction

Pressure ulcers (PU) usually occur on bony prominences as a result of pressure or a combination of pressure and shear and/or friction [1] and are common among coma, paralyzed, or bedridden patients, as well as patients with diabetes and weak constitution [2]. PU pose a serious threat to health and increase the economic burden on patients and society [3]. The difficulty in treating PU and the recurrence of PU have been bothering clinicians for a long time.

PU healing is often difficult to achieve due to vasculopathy, inflammatory response, and tissue necrosis caused by the long-term pressure on local skin tissues [4, 5], as well as hypoxia, malnutrition, and ischemic necrosis [6]. Current treatments for PU include relieving the pressure and protecting the wounds with dressings [7], preventing infection and performing debridement to reduce damage, and stimulating cell proliferation/migration and angiogenesis to accelerate wound healing [8–12]. Research concerning PU treatment has focused on antibacterial therapy, wound acidification, and enhancement of angiogenesis and microcirculation [13]. The promotion of damaged blood vessel repair plays an important role in the treatment of skin PU. Nonetheless, few treatments can effectively promote damaged blood vessel repair and rapid angiogenesis at the injured site of PU.

Vascular endothelial cells (VECs) in the skin and subcutaneous tissues are in a state of hypoxia when PU occur, which diminishes cell proliferation [14]. Moreover, hypoxia can cause ferroptosis [15, 16]. We speculate that the VECs in PU tissues may undergo ferroptosis. Therefore, inhibiting the ferroptosis of VECs under hypoxia may be of great importance in repairing injured blood vessels and promoting wound healing. Milk fat globule-epidermal growth factor 8 (MFGE8), a secretory protein that can be encapsulated in exosomes [17], promotes angiogenesis, inhibits inflammation in cutaneous ischemia–reperfusion injury and also accelerates skin wound healing by regulating cell proliferation [18–22]. However, no study has investigated whether MFGE8 can inhibit blood vessel injury and repair skin PU via inhibition of the ferroptosis of VECs under hypoxia. Previous studies reported that MFGE8 was significantly decreased in PU tissues [20, 23], suggesting that MFGE8 might be an effective factor for accelerating wound healing.

Extracellular vesicles (EVs) have been recognized as a promising drug delivery system and therapeutic tool [24, 25], such as in fighting with neurodegeneration [26], delaying intervertebral disc degeneration [27] and treating intestinal disease [28]. However, natural EVs are not only small in quantity but also contain few bioactive proteins; thus, obtaining a large amount of EVs for disease treatment may not be possible. Therefore, in this study, we considered whether the key proteins in EVs could be purified by Chinese hamster ovary (CHO) cells and coated with silk fibroin nanoparticles (NPs) to obtain a large number of EV-derived NPs. Considering that the MFGE8 protein can be contained in EVs [17] and acts as an effective factor for accelerating wound healing, silk fibroin NPs are used to prepare NPs@MFGE8 via coating MFGE8 to carry and deliver MFGE8 to VECs in PU tissues and analyze the role of MFGE8 in promoting PU, which may serve as an effective therapeutic approach for PU.

During neovascularization, aminopeptidase N (CD13) is usually highly expressed on the surface of VECs within tumors [29, 30] or granulation tissues [31, 32] in hypoxic environments, promoting granulation tissue hyperplasia and tumor growth. Nonetheless, whether VECs overexpress CD13 in hypoxic PU tissues remains unclear. NGR, a polypeptide containing arginine, glycine, and asparagine sequences, is a ligand with high affinity for CD13 [33]. In previous studies, the NGR peptide was combined with anti-tumor drugs to construct anti-tumor drugs targeting tumor neovascularization [34, 35]. Therefore, studying the expression of CD13 in skin PU and designing drug-loaded, cross-linked NGR NPs may enable the accurate targeting of VECs in PU tissues, consequently accelerating wound healing more effectively.

In the present study, we identified that the inhibitory effect of EVs on ferroptosis was mainly carried out through MFGE8. EV-derived NPs@MFGE8 was then prepared by NPs coating MFGE8. For targeted treatment of PU, NGR-NPs@MFGE8 was assembled via cross-linking NPs@MFGE8 with the NGR peptide, which could target CD13 and release MFGE8 at the site of PU. Then, silk fibroin and collagen were used to synthesize the silk fibroin/collagen hydrogel. After being loaded with NPs, NPs@MFGE8 or NGR-NPs@MFGE8, silk fibroin/collagen hydrogel sustained-release carrier was synthesized. When applied to the treatment of PU, silk fibroin/collagen hydrogel sustained-release carrier loaded with NGR-NPs@MFGE8 could not only absorb wound exudates but also release NGR-NPs@MFGE8 to target CD13 located on the surface of VECs for a long time compared with those loaded with NPs and NPs@MFGE8, inhibiting ferroptosis due to hypoxia and promoting wound healing. The present study provides a novel method for the effective treatment of skin PU.

## Materials and methods

### Reagents and antibodies

3-Methyladenine (cat. no. S2767), an autophagy inhibitor, was purchased from Sellect (Shanghai, China). Dimethyl sulfoxide (cat. no. 67-68-5) was supplied by Sigma (St. Louis, Missouri, USA). Erastin (cat. no. HY-15763), a ferroptosis inducer, was obtained from MedChemExpress (Shanghai, China). Rhodamine B isothiocyanate (RBITC; cat. no. GY418) was obtained from Goyoo Biotech (Nanjing, China). Collagenase type II (cat. no. A004174), N-hydroxysuccinimide (NSH) (cat. no. 6066-82-6), and 1-ethyl-3-(3-dimethylaminopropyl) carbodiimide hydrochloride (EDC) (cat. no. 25952-53-8) were obtained from Sangon Biotech (Shanghai, China). PKH26 (cat. no. MINI26) and collagenase D (cat. no. 11088858001) were provided by Sigma (St. Louis, Missouri, USA). NGR peptide (cat. no. GC14926) was purchased from GlpBio Technology (Montclair, California, USA). HOOC-PEG-COOH (cat. no. R-1202-1 k) was purchased from RuiXi Biological Technology (Xi'An, China). Anti-glyceraldehyde 3-phosphate dehydrogenase (cat. no. 60004-1-Ig), anti-P53 (cat. no. 10442-1-AP), anti-LC3A/B (cat. no. 14600-1-AP), anti-P62 antibody (cat. no. 18420-1-AP), anti-GPX4 antibody (cat. no. 67763-1-Ig), anti-LC3B antibody (cat. no. 18725-1-AP), anti-CD13 antibody (cat. no. 14553-1-AP), anti-CD31 antibody (cat. no. 66065-2-Ig), anti-pink1 antibody (cat. no. 23274-1-AP), anti-parkin antibody (cat. no. 66674-1-Ig), anti-CD63 antibody (cat. no. 25682-1-AP), anti-CD14 antibody (cat. no. 17000-1-AP), and anti-α-SMA antibody (cat. no. 14395-1-AP) were obtained from Proteintech (Wuhan, China). Anti-TSG101 antibody (cat. no. A1692) was obtained from ABclonal Technology (Wuhan, China). Anti-ACSL4 antibody (cat. no. ab155282), goat anti-mouse IgG antibody (Alexa Fluor^®^ 488) (cat. no. ab150113), and goat anti-rabbit IgG antibody (Alexa Fluor^®^ 594) (cat. no. ab150080) were provided by Abcam (Cambridge, Massachusetts, USA). Immunol Fluorescence Staining Kit with Alexa Fluor 647-labeled goat anti-rabbit IgG (cat. no. A0468), DAPI nucleic acid dye (cat. no. C1006), and crystal violet staining solution (cat. no. C0121) were provided by Beyotime Biotechnology (Shanghai, China). Anti-MFGE8 (cat. no. sc-8029) was provided by Santa Cruz Biotechnology (Santa Cruz, USA).

### Patients

Instead of necrotic tissues, mild and/or severe PU tissues were acquired from each patient who underwent skin debridement surgery. A total of thirteen tissues (mild ulcer tissues, *n* = 7; severe ulcer tissues, *n* = 6) were collected. Mild and severe PU tissues were used for western blotting as well as immunohistochemical and immunofluorescence staining. This study received official approval from the Ethics Committee of the Second Affiliated Hospital of Chongqing Medical University (approval number: 2022-KLSD-198). Written informed consent was obtained from all patients or their relatives prior to tissue collection.

### Isolation of VECs

Two-week-old rats were anesthetized with 3% pentobarbital sodium (50 mg/kg) and sacrificed with a broken neck. The abdominal skin was cut open to expose the abdominal blood vessels. A section of the blood vessel was cut, the whole vessel was turned over to expose the vascular endothelium, and both ends of the vessel were ligated and digested with 2% collagenase D and 2% collagenase type II at 37 °C for 1–2 h. The supernatant was collected after digestion and was subsequently centrifuged at 1200 rpm/min for 5 min. Cell precipitates were collected, resuspended in a complete medium, and inoculated into culture dishes. Cell culture and identification of VECs were performed after cell fusion to 80–90%.

### Hypoxic culture of VECs

VECs that reached 90–100% confluence were digested with trypsin and then expanded three times for culture. Normal control cells were cultured with 21% O_2_ and 5% CO_2_ at 37 °C. The hypoxic culture of VECs was cultured in a hypoxic incubator (Whitley H35 Hypoxystation; Don Whitley, UK) under the following conditions: 1–3% O_2_, 5% CO_2_, and 37 °C. Then, the cells were collected for gene sequencing (Novogene, Beijing, China), flow cytometry, and western blotting.

### Extraction, identification, and mass spectrometric detection of EVs

VECs that reached 90–100% confluence were digested with trypsin and then expanded for culture. When the cells reached 80–90% confluence, further culture was carried out for 3–4 d using serum-free DMEM/F12 medium, and the supernatant was collected after culture. The culture medium was centrifuged at 300 ×*g* for 10 min, 1000 ×*g* for 10 min, and 10,000 ×*g* for 30 min to collect the supernatant and finally at 100,000 ×*g* for 70 min to collect the EV precipitates. EVs were resuspended in 200 μL of phosphate-buffered saline (PBS). Subsequently, 100 μL of suspension EVs was taken out and mixed well with 5 μL of PKH26 dye in 300–500 μL of diluent in the dark for 5–10 min, and 500 μL of 5% BSA was then added to terminate the reaction. PKH26-labeled EVs (PKH26-EVs) were obtained by centrifugation at 100,000 ×*g* for 70 min. The particle size of EVs was detected using a zeta particle size analyzer. The characteristic protein of EVs was detected by western blotting, and the morphology of EVs was observed by transmission electron microscopy (TEM). EVs were examined by mass spectrometry at the Central Laboratory of the Army Medical University to detect the protein species.

### Lentivirus transfection

To overexpress MFGE8, the lentivirus was obtained from HanBio (Shanghai, China). VECs were inoculated and cultured in six-well plates. Diluted Lenti-MFGE8 or Lenti-NC and 1 μL Polybrene (2 μg/mL) were added when the VECs reached 50% confluence. After shaking evenly, the VECs were cultured in an incubator at 37℃ and 5% CO_2_. After 24 h, the medium was changed, and the VECs continued to culture for 3–5 d. After the VECs were treated with puromycin (10 μg/mL) for 5–7 d, the transfection efficiency of the VECs was analyzed by western blotting.

### Western blotting

After grinding and crushing, the VECs or skin tissues were lysed with RIPA (cat. no. P0013B) and PMSF (cat. no. ST506) from Beyotime Biotechnology (Shanghai, China). After being lysed for 30 min and centrifugated at 12,000 rpm/min for 10 min, the supernatant was collected and added with 5 × SDS-PAGE sample loading buffer (cat. no. P0015, Beyotime Biotechnology, Shanghai, China). When the samples were electrophoresed at the bottom of PAGE Gel at 120 V for 70 min, the protein samples were transferred to polyvinylidene fluoride (PVDF) at 300 mA for 70–120 min. PVDF was sealed with 5% degreased milk powder for 1 h. After washing, PVDF was incubated with the diluted first antibody overnight at 4 °C. On the second day, after washing, PVDF was incubated with the diluted second antibody (1:5000) at 20–30 °C for 1–2 h. After washing, the High Sensitivity ECL Kit was used to formulate the luminescent solution (A:B/1:1) and to image the PVDF in an imaging system.

### Immunofluorescence and immunohistochemistry

Paraffin-embedded tissue sections were dewaxed to hydrate. The samples were then subjected to immunofluorescence or immunohistochemistry. Tissue samples were repaired with trypsin antigen repair solution and endogenous peroxidase blocker. After washing, the tissue samples were sealed with 5% BSA for 30 min and then incubated with dilute rat or rabbit first antibody at 4 °C overnight. After incubation, they were washed with PBS. In immunofluorescence experiments, the secondary antibodies of goat against rabbit or rat were diluted, and the samples were incubated at 37 °C for 1–2 h. Immunofluorescence staining was performed for 5 min using a DAPI staining solution, and the samples were then photographed under a fluorescence microscope or laser confocal microscope. In immunohistochemical tests, HRP-labeled secondary antibody was incubated with tissues at 37 °C for 30 min. After washing, the samples were stained with DAB solution and observed under a microscope; additionally, they were stained with hematoxylin for 5 min. After dehydration and fixation, the tissue sections were observed and photographed using the M8 digital scanning microscopic imaging system.

### Purified protein extraction

CHO cells were cultured in incubators under static conditions with 5% CO_2_ at 37 °C and then transfected with 3xFlag-labeled MFGE8-PURO lentivirus. After 3 d, the CHO cells were digested with trypsin after fusion to 80–100%. After treating with puromycin (10 μg/mL) for 5–7 days under static conditions, the CHO cells were digested and centrifuged at 1000 rpm/min for 5 min. Subsequently, the CHO cells were cultured in suspension in a shaker at a rotating speed of 80–100 rpm/min with the RAPID CHO 18 serum-free medium (cat. no. H180KJ, BasalMedia, Shanghai, China). After incubation for 48–72 h, the suspended cells were centrifuged, and CHO cell precipitates were collected. The CHO cells were lysed with RAPI and protease inhibitor PMSF for 30–60 min. Subsequently, ultrasound was performed under the following conditions: ultrasonic time, 20–30 s; amplitude, 20%; pause, 2 s; and ultrasound, 2 s. After centrifuging, the protein supernatant was collected and added to a 20-μL anti-Flag magnetic bead suspension according to the 500-μL protein supernatant. Magnetic beads were separated by a magnetic frame for 30 s following incubation for 2 h at 20–30 °C in a shaker. After being washed with TBS buffer two times, the 20 μL magnetic bead suspension was incubated with 100 μL of 3xFlag polypeptide for elution at 25 °C for 30–60 min. The magnetic beads were then separated by a magnetic frame for 30 s to collect the eluent containing purified MFGE8 protein.

### Synthesis and characterization of NPs

Cocoons (2 g), provided by the State Key Laboratory of Resource Insects, Southwest University, Chongqing, were taken and placed into 1 L of water. Afterwards, 5–10 g of Na_2_CO_3_ was added so that the Na_2_CO_3_ concentration was 0.5–1%. These cocoons were then boiled in 90–100 °C water for 30–60 min. A dissolved solution (50 mL) was prepared with deionized water (30.43 mL), ethanol (19.57 mL), and calcium chloride (24 g). The cocoons were dissolved at 60 ± 5 °C for 1–2 h until complete dissolution of silk fibroin. Centrifugation was performed at 10,000 ×*g* for 10 min, and the supernatant was collected. The silk fibroin solution was filtered for 48 h in a dialysis bag with an interception molecular weight of 8000–14000 Da. After filtration, the solution was centrifuged at 10,000 ×*g* for 10 min. The obtained silk fibroin solution was diluted to 20 mg/mL (2%). Then, 5 mL of acetone solution was added to a 15-mL centrifuge tube, 1 mL of 1% silk fibroin solution was added to the acetone solution by drops, and vortexing was continued for 30–60 s. Then, ultrasound was conducted on ice under the following conditions: ultrasonic time, 2 min; pause, 2 s; ultrasound, 2 s; and amplitude, 30%. Then, the milky supernatant was obtained by centrifugation at 3840 × *g* for 5 min. The milky supernatant was centrifuged at 10,629 × *g* for 10 min to obtain no-load NPs. For obtaining the NPs@MFGE8, approximately 200 μl of purified MFGE8 protein (1 mg/ml) was added to 200 μl of silk fibroin solution (10 mg/ml or 5 mg/ml) with a mass ratio of 1:10 or 1:5. After swirling well, the mixed solution was added to the acetone solution (2–3 ml) by drops and then swirled in a vortex mixer for 30–60 s. Ultrasound was conducted on ice under the following conditions: ultrasonic time, 2 min; pause, 2 s; ultrasound, 2 s; and amplitude, 30%. Then the milky supernatant was obtained by centrifugation at 1699 ×*g* for 5 min. The milky supernatant was centrifuged at 10,629 ×*g* for 10 min to obtain MFGE8-coated NPs (NPs@MFGE8). Afterwards, 10 mL of deionized water was added for washing, centrifugation was performed at 10,629 ×*g* for 10 min, the supernatant was poured off to collect the precipitates, and the acetone residue was removed. NPs@MFGE8 was stored at 4℃ for short-term use within 3 days and at − 80 ℃ for long-term use within 6 months, and repeated freezing and thawing were avoided. The particle size and potential of NPs and NPs@MFGE8 were detected using a zeta potential and particle size analyzer. RBITC (10 mg) was dissolved in deionized water (1 mL), and 5 μL of 1% RBITC staining solution was added to 1 mL of resuspended NP solution. RBITC-labeled NPs (RBITC-NPs) were obtained after the reaction transpired for 30 min under a light shield at 20–30 °C.

### Characteristics of NPs clearance after the uptake by cells

After treatment with RBITC-NPs or RBITC-NPs@MFGE8, the mean fluorescence intensity (MFI) of VECs was statistically analyzed at 0, 1, 3, 5 and 7 days. MFI-0 was the mean fluorescence intensity at day 0. MFI-x was the mean fluorescence intensity at day 1, 3, 5 or 7. The clearance rate was calculated as follows: (%) = (MFI-0—MFI-x)/MFI-0 × 100%.

### Synthesis and characterization of NGR-NPs@MFGE8

NPs or NPs@MFGE8 produced from 500 μL of 1% silk fibroin solution or/and 500 μL of purified MFGE8 protein (1 mg/mL) solution were dissolved in 1 mL of deionized water. EDC (5 mg), HOOC-PEG-COOH (10 mg), and NGR peptide (10 mg) were added to NPs solution (1 mL) and allowed to react at 20–30 °C for 0.5–1 h in a shaker. Subsequently, NSH (5 mg) was added and incubated overnight at 20–30 °C in a shaker. On the second day, the solution was centrifuged at 1699 ×*g* for 5 min, and the supernatant was discarded to obtain cross-linked NGR NPs (NGR-NPs) or cross-linked NGR NPs@MFGE8 (NGR-NPs@MFGE8) precipitate. The particle size and zeta potential of NGR-NPs and NGR-NPs@MFGE8 were detected using a zeta potential and particle size analyzer (Thermo Fisher Scientific, Massachusetts, USA). Fourier transform infrared spectrometry (FT-IR) was conducted for NPs, NPs@MFGE8, NGR-NPs, and NGR-NPs@MFGE8.

### Envelopment efficiency assay

High-performance liquid chromatography (HPLC) was performed to measure the envelopment efficiency. First, the total amount of MFGE8 protein was detected using an ultra-fine ultraviolet spectrophotometer (NanoDrop OneC, Thermo Fisher Scientific, Massachusetts, USA) and divided into two equal fractions (namely, fractions A and B) before coating. Fraction A of the purified protein was used for NPs@MFGE8 according to the amount of silk fibroin protein and MFGE8 (10:1 or 5:1). After obtaining the precipitated NPs@MFGE8 by centrifugation, NPs@MFGE8 were lysed with lithium bromide (LiBr) (9.3 mol/L). Then, HPLC was conducted to detect the chromatogram and quantity of free MFGE8 in NPs@MFGE8 and fraction B of the purified MFGE8 protein. The envelopment efficiency was then calculated from the ratio of free MFGE8 to the total quantity of MFGE8 in fraction B.

### Characteristics of MFGE8 released from NPs@MFGE8

The characteristics of MFGE8 released from NPs@MFGE8 were detected by HPLC. On day 0, we obtained 800 μl of MFGE8 solution (1 mg/ml), added the MFGE8 solution to 800 μl of silk fibroin solution (10 mg/ml), and prepared the NPs@MFGE8 solution after mixing evenly. The NPs@MFGE8 solution was divided equally into eight aliquots of 200 μl each. One aliquot was centrifuged at 10,629 ×*g*, and the NPs@MFGE8 precipitate was collected. NPs@MFGE8 was lysed with 9.3 mol/L LiBr, and the total content of MFGE8 (Mt) was determined by HPLC. The remaining 7 portions were rotated and shaken in a shaker (20 rpm/min) at 37 ℃. Then on days 1, 2, 3, 4, 5, 6, and 7, the NPs@MFGE8 solution was centrifuged at 10,629 ×*g*, the supernatant was collected, and the release content of MFGE8 (Mr) in the supernatant was detected by HPLC. The release rate was calculated as follows: (%) = Mr/Mt × 100%.

### Synthesis and characterization of collagen, silk fibroin, and silk fibroin/collagen hydrogels

Collagen hydrogel was prepared from rat tail collagen. One rat was anesthetized with pentobarbital sodium (50 mg/kg) and subsequently sacrificed by breaking its neck. The tail was removed and washed with PBS. It was then cut through the skin, exposing the white collagen of the tail. The tail collagen was removed, cut to a size of 1–3 mm^3^, and washed with PBS two to three times. The collagen was centrifuged at 4000 rpm/min (3220 ×*g*) for 5 min, and the supernatant was poured away. The collagen precipitate was added to 100 mL of 0.5–1% (v/v) acetic acid solution. The collagen was dissolved by shaking at 4 °C for 48 h in a shaker and, after complete dissolution, was centrifuged at 12,000 ×*g* for 10 min. The supernatant was collected and then stirred and salted out for 5 min in 10% NaCl solution, after which a large amount of flocculent collagen was precipitated. The flocculent collagen was centrifuged at 12,000 ×*g* for 10 min at 4 °C to collect the collagen. To fully dissolve the collagen, 50–100 mL of 0.1 mmol/L HCl solution was added to the collagen. Additionally, 3 mL of 0.5% collagen solution was added to approximately 240 μL of 1 mol/L NaOH so that the pH value reached approximately 7.0. Collagen hydrogel was formed at 37 °C for 5–10 min, whereas silk fibroin hydrogel was prepared from the silk fibroin solution. Carbomer (0.24 g) was dissolved in 1 mL of deionized water and stirred well. The carbomer solution was mixed with 800 μL of 1 mol/L NaOH solution and stirred well. Afterwards, 800 μL of 20% polyvinyl alcohol was added. Subsequently, 4 mL of 6% silk fibroin solution was added to the carbomer solution, stirred well, and placed at 20–30 °C for 12–24 h until it cross-linked with the silk fibroin hydrogel. Silk fibroin/collagen hydrogel was prepared from rat tail collagen and silk fibroin solution. Carbomer (0.24 g) was dissolved in 1 mL of deionized water and stirred well. Furthermore, 800 μL of 1 mol/L NaOH solution was added to the carbomer solution and stirred well. Subsequently, 800 μL of 20% polyvinyl alcohol was added, and 4 mL of 6% silk fibroin solution was added to the carbomer solution and stirred well. Afterwards, 4 mL of 0.5–1% collagen solution was mixed evenly with about 320 μL of 1 mol/L NaOH solution, then added to the carbomer solution and stirred well, and placed at 20–30 °C for 12–24 h until the silk fibroin/collagen hydrogel formation. To prepare the silk fibroin/collagen hydrogel loaded with NPs, the prepared NPs were added to the uncrosslinked silk fibroin/collagen hydrogel. The silk fibroin/collagen hydrogel loaded with NPs was stirred thoroughly, filtered several times with a 70 μm filter by centrifugation at 3840 ×*g* to make its distribution uniform, and then placed at 20–30 °C for 12–24 h until the silk/collagen hydrogel loaded with NPs formation. Scanning electron microscopy (SEM) and rheometer were used to detect the void size and the energy storage modulus (G′) and loss modulus (G″) of hydrogels, respectively. The swelling rate was measured at different time points. The weight of the dried hydrogel was determined, and then the dried hydrogel was immersed in PBS solution at 37 ℃. The hydrogel soaked in PBS at the set time point was taken out, the water on the surface was quickly removed, and the hydrogel was weighed. W_s_ was the weight of the hydrogel after swelling, and W_d_ was the weight of the hydrogel after drying. The swelling ratio was calculated as follows: (%) = (W_s_−W_d_)/W_d_ × 100%.

### Release assay of NPs in silk fibroin/collagen hydrogel

NPs were labeled with 1% RBITC (RBITC-NPs), with an absorption wavelength of approximately 550 nm. The NPs release capacity of silk fibroin/collagen hydrogel was characterized by analyzing the in vitro release of RBITC-NPs from the hydrogel. Briefly, the absorbance of gradient dilutions of RBITC-NPs with PBS at 550 nm was used to draw a standard curve for RBITC-NPs. RBITC-NPs loaded silk fibroin/collagen hydrogel was suspended in 4 mL of PBS in a centrifuge tube that was rotated at 37 °C. At various time points, supernatants were collected, and tubes were replenished with the same volume of PBS. The integrated absorbance of RBITC-NPs released from the silk fibroin/collagen hydrogel was determined using the iMark™ Microplate Reader (Bio-Rad, USA). The cumulative release of RBITC-NPs was calculated with the help of a standard curve.

### Biocompatibility assay

VECs were inoculated and cultured in six-well plates. Diluted Lenti-NC (1:50) and 1 μL Polybrene (2 μg/mL) were added when the VECs reached 50% confluence. Then puromycin (10 μg/mL) was added and continued to culture for 3–5 d until 80–90% or more cells appeared green. The silk fibroin/collagen hydrogels were synthesized and soaked in complete medium for 24 h. The medium was changed every 6 h until the color of the medium did not change. The silk fibroin/collagen hydrogel was cut into disks with a thickness of 1–2 mm and a diameter of 1 cm and stained with RBITC for 2 h. 100 μM of 1 × 10^5^ VECs transfected with Lenti-NC were inoculated and cultured on silk fibroin/collagen hydrogels. Two hours later, 1 mL of complete medium was added. On the second day, the hydrogel was removed and imaged using a microscope (Olympus, Japan).

### Degradation of the hydrogel carrier assay

Collagenase I and II, trypsin and neutral proteinase were used to degrade hydrogel carriers for 10 days. On day 0, the mass of the dry hydrogel carrier was weighed as H0, and then on days 1, 3, 5, 7, and 10, the mass of the dry hydrogel was weighed as Hm. The degradation rate of the hydrogel was calculated as follows: (%) = (H0−Hm) /H0 × 100%.

### Flow cytometry

The mitochondrial membrane potential of VECs was detected using the Mitochondrial Membrane Potential Detection Kit (cat. no. E-CK-A301) from Elabscience Biotechnology (Wuhan, China). Cells were digested with trypsin for 1–2 min, and a complete medium was added to terminate the digestion. The cell suspension was collected and centrifuged at 300 ×*g* for 5 min. The cells were resuspended in 500 μL of JC-1 working solution and incubated at 37 °C for 20 min. After incubation, the cells were centrifuged at 300 ×*g* for 5 min. The cell precipitates were resuspended using 1xJC-1 assay buffer and then assayed by flow cytometry (Beckman, USA). Hypoxia-induced cells were collected and stained using an apoptosis kit (cat. no. PI0100, Invigentect, USA). Propidium iodide staining was performed for 5 min. The death rate was then determined by flow cytometry.

### Cell viability and proliferation assay

VECs that reached 90–100% confluence were digested and then inoculated into 96-well plates. The cell viability was tested using the Cell Counting Kit-8 (cat. no. BG0025, BG Biotech Beijing, China). After processing the different groups, 10 μl of CCK-8 reagent was added, and the cells were incubated at 37 ℃ in the dark for 2–5 h. The absorbance of the medium for each culture well was measured at a wavelength of 450 nm, and the cell viability level was statistically analyzed. Moreover, the proliferation of VECs was also measured using 5-ethynyl-2′-deoxyuridine (EdU) (cat. no. C10310-3, RiboBio, Guangzhou, China). After 12 h of incubation with Edu solution, Apollo staining solution was added to the VECs for incubation for 30 to 60 min. The VECs were then washed with PBS and subjected to DNA staining. Fluorescence images were obtained by fluorescence microscopy (Olympus, Japan).

### TEM

VECs or EVs were collected and fixed overnight with 3% glutaraldehyde. After washing, they were fixed with 1% osmium acid for 2 h. Subsequently, the samples were subjected to gradient dehydration with different acetone concentrations. After the samples were embedded, ultrathin sections (70–90 nm) were prepared and stained with 5% uranium and lead citrate. Finally, the samples were observed using TEM (Philips, Amsterdam, The Netherlands).

### SEM

Silk fibroin or collagen was used in preparing the hydrogel or NPs. Droplets of NPs were added to silicon wafers and naturally dried at 25 °C. The hydrogel was freeze-dried overnight. On the second day, the dried hydrogel was taken out, soaked in liquid nitrogen for 1–2 min, and cut into 1–2-mm thin slices. The NPs and hydrogel were treated with gold spray, and their structure and morphology were then observed via SEM.

### Lipid peroxidation and reactive oxygen species (ROS) assay

Lipid peroxidation and ROS levels were analyzed using C11-BODIPY™ 581/591 (cat. no. D3861, Thermo Fisher Scientific, Massachusetts, USA) or CellROX™ Deep Red Flow Cytometry Assay Kit (cat. no. C10491, Thermo Fisher Scientific, Massachusetts, USA). Adherent cells were taken and washed with PBS. The cells were digested with trypsin for 1–2 min. After 30–50% of cells were digested, a complete medium was added to terminate the digestion. The cell suspension was collected and centrifuged at 300 ×*g* for 5 min. The cells were resuspended with C11-BODIPY™ 581/591 or CellROX™ Deep Red reagent at an appropriate concentration and were then incubated at 37 °C for 30–60 min away from light. Afterwards, the cells were washed with PBS and analyzed using flow cytometry.

### Glutathione (GSH) assay

GSH levels were detected using the GSH Colorimetric Assay Kit (cat. no. E-BC-K030-M) from Elabscience Biotechnology (Wuhan, China). The VECs were harvested by cell scraping and washed once or twice with PBS, after which ultrasound was performed on ice. After centrifuging at 1500 ×*g* for 10 min, the cell supernatant was incubated with 100 μL of acid reagent at 20–30 °C and then centrifuged at 4500 ×*g* for 10 min. The protein concentration of the supernatant was determined using a BCA kit (cat. no. P0012S, Beyotime Biotechnology, Shanghai, China). Subsequently, 25 μL of 5,5′-dithiobis(2-nitrobenzoic acid) solution was added to a 96-well plate, along with 100 μL of the above mentioned supernatant, which was incubated for 5 min at 20–30 °C. A microplate reader was then used to measure the absorbance values of each well at 405–414 nm. The amount of GSH in the cells was calculated based on the GSH standard curve and normalized to the protein concentration.

### Malondialdehyde (MDA) assay

MDA levels of VECs were detected by the MDA Colorimetric Assay Kit (cat. no. E-BC-K028-M) from Elabscience Biotechnology (Wuhan, China). The VECs were harvested by cell scraping and washed once or twice with PBS. For ultrasound on ice, an extracting solution (0.5 mL) was added to the cell suspension. A 0.1 mL sample was mixed with 1 mL working solution and incubated in 100 °C water for 40 min. After cooling to 20–30 °C, the samples were centrifuged at 1078 ×*g* for 10 min. The protein concentration was measured using a BCA kit (cat. no. P0012S, Beyotime Biotechnology, Shanghai, China). The supernatant (250 μL) was added to a 96-well plate, and a microplate reader was then used to measure the absorbance values at 532 nm. The MDA level in each sample was normalized to the protein concentration.

### Iron assay

Intracellular iron in VECs was measured by the Iron Colorimetric Assay Kit (cat. no. E1042) from Applygen (Beijing, China). The VECs cultured in 24-well plates were washed with cold PBS, and 200 μL of lysis buffer was then added to each well and lysed for 2 h in a shaker. Working solution A was prepared by mixing the buffer with 4.5% potassium permanganate solution at a 1:1 ratio. Next, 100 μL of working solution A and 100 μL of the sample were mixed well and incubated at 60 °C for 1 h. After cooling to 20–30 °C, 30 μL of iron ion agent was added to the sample, mixed well, and incubated at 20–30 °C for 30 min. The supernatant (200 μL) was added to a 96-well plate, and a microplate reader was used to detect the absorbance at 550 nm. The iron levels were normalized to the protein concentration.

### Animal experiments

The 3-week-old rats (about 60–80 g) were provided by the Animal Center, Army Medical University, and anesthetized with 3% pentobarbital sodium (50 mg/kg). After hair removal, pressure was applied using cylindrical magnets with a diameter of 8 mm and height of 3 mm, which was continued for 6–8 h daily until ulcers formed. 10 rats were used to observe the diffusion of RBITC-NPs in vivo. Silk fibroin/collagen hydrogel loaded with RBITC-NPs was applied to prepare hydrogel sustained-release carrier. Necrotic tissues were removed, and the hydrogel sustained-release carrier was fixed on the ulcer surface with a thin film. The treatment was continued by changing the hydrogel daily for 5 d. In vivo imaging and fluorescence microscopy were performed to observe the diffusion of RBITC-NPs in PU skin tissues at days 0, 1, 3, 5, and 7.

Six rats were divided into the normal control (NC) and PU groups to evaluate whether RBITC-labeled NGR-NPs@MFGE8 (RBITC-NGR-NPs@MFGE8) could target VECs in PU tissues. The NC group was not damaged with PU; however, the surface skin structure was partially removed using a blade to ensure that RBITC-NGR-NPs@MFGE8 had crossed the barrier between the skin and subcutaneous tissues. Subsequently, the skin in both the NC group and the PU group was treated with RBITC-NGR-NPs@MFGE8-loaded hydrogel and observed under fluorescence.

To assess the healing effect of hydrogel sustained-release carrier on the ulcer surface, 24 rats were divided into six groups—namely, the NC (*n* = 3), PU (*n* = 3), PU + silk fibroin/collagen hydrogel (Gels) (*n* = 6), PU + silk fibroin/collagen hydrogel-NPs (Gels-NPs) (*n* = 3), PU + silk fibroin/collagen hydrogel-NPs@MFGE8 (Gels-NPs@MFGE8) (*n* = 6), and PU + silk fibroin/collagen hydrogel-NGR-NPs@MFGE8 (Gels-NGR-NPs@MFGE8) (*n* = 3) groups. Necrotic tissues were removed, and the Gels, Gels-NPs, Gels-NPs@MFGE8 or Gels-NGR-NPs@MFGE8 were fixed on the ulcer surface with a thin film, respectively. The ulcer surface was then photographed at days 0, 1, 3, 5, and 10. After treatment, the blood perfusion of PU tissues was statistically analyzed, and PU tissues were used for Masson staining, immunohistochemistry staining, immunofluorescence staining, and electron microscopy observation of mitochondrial structure changes in VECs. Animal use and experimental procedures were given official approval by the Animal Ethics Committee of the Army Medical University (no. AMUWEC20230372).

### Statistical analyses

The data are presented as means ± standard deviations on at least three measurements and were analyzed by Student’s t-test or one-way analysis of variance using SPSS version 22.0 (IBM Corp., NY, USA) or GraphPad Prism version 7.0 (GraphPad Software Inc., CA, USA). Graph analysis was performed using GraphPad Prism version 7.0. Statistical significance was set at *p* < 0.05.

## Results

### Ferroptosis of VECs occurred in PU tissues under hypoxia

**Fig. 1:**
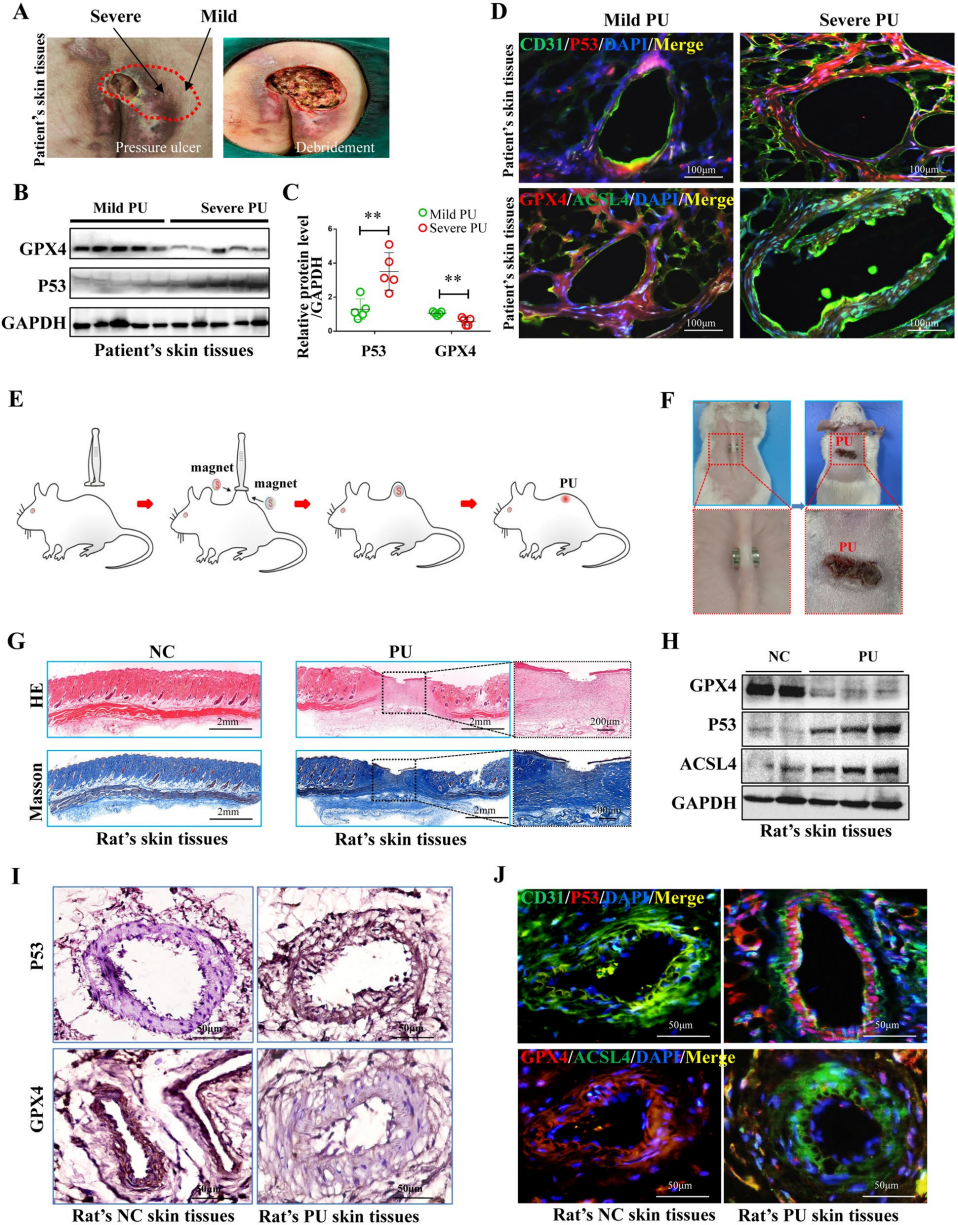
Detection and analysis of ferroptosis-related proteins in rats’ and patients’ PU tissues. **A** Mild and severe PU tissues were collected from patients who underwent debridement. **B**, **C** Representative western blotting and quantitative levels of GPX4 and P53 in patients with mild and/or severe PU. **D** Representative immunofluorescence staining of CD31/P53 and GPX4/ACSL4 in VECs in mild and severe PU tissues from patients. **E** Model construction method for PU in rats. After the rats were anesthetized, the skin hair at the back of rats was removed, and the skin was then lifted and pressurized using cylindrical magnets. The pressure was continued to be applied for 6–8 h daily until PU formed. **F** Cylindrical magnets with a diameter of 8 mm and height of 3 mm were applied on the back skin to form PU in rats. **G** H&E staining and Masson staining of normal and PU skin tissues in rats. **H** Representative western blotting of GPX4, P53, and ACSL4 in normal and PU skin tissues in rats. **I**, **J** Representative immunohistochemical staining of P53 and GPX4 and immunofluorescence staining of CD31/P53 and GPX4/ACSL4 in blood vessels in normal and PU skin tissues in rats. NC: normal control, PU: pressure ulcer. ns: *p* > 0.05; *p < 0.05; **p < 0.01; ***p < 0.001

To clarify whether the occurrence of PU was related to vascular injury and ferroptosis of VECs caused by continuous hypoxia, mild and severe PU tissues were obtained from patients undergoing skin debridement surgery (Fig. 1A). Subsequently, the expression of ferroptosis-related proteins in PU tissues was detected by western blotting. Mild PU tissues exhibited higher levels of the anti-ferroptosis-related protein GPX4 and lower levels of the promoting ferroptosis-related protein P53 than severe PU tissues (Fig. 1B). Additionally, the relative protein expression levels of GPX4 and P53 were statistically different in mild and severe PU tissues (Fig. 1C). As observed with immunofluorescence staining of blood vessels in PU tissues, mild PU tissues showed lower P53 and ACSL4 expression and higher GPX4 expression in VECs than severe PU tissues (Fig. 1D). In order to verify the correlation between PU and ferroptosis of VECs in rats, we constructed a rat model of PU, as shown in Fig. 1E. After anesthetizing the rats with 3% pentobarbital sodium (50 mg/kg), pressure was applied using cylindrical magnets with a diameter of 8 mm and height of 3 mm, which was continued for 6–8 h daily until PU formed (Fig. 1F). H&E staining and Masson staining were performed on the NC and PU skin tissues. The results suggested that the rat PU model could be effectively constructed by employing the magnet compression method (Fig. 1G). The association between ferroptosis and PU was subsequently verified by western blotting and immunohistochemical staining in rats. The difference in the expression of ferroptosis-related proteins GPX4/P53/ACSL4 in NC and PU skin tissues indicated that ferroptosis was positively correlated with the occurrence of PU (Fig. 1H–I). Moreover, immunofluorescence staining of the VECs marker protein CD31 and ferroptosis-related proteins suggested that GPX4 decreased, whereas P53 and ACSL4 increased in VECs after PU formation (Fig. 1J). Considering that the ferroptosis of VECs might be an important cause of PU, we extracted and identified VECs from rats. Immunofluorescence staining (Additional file 1: Fig. S1A) and flow cytometry (Additional file 1: Fig. S1B) were conducted to detect the VECs marker protein CD31, and the results indicated that the primary cells highly expressed CD31, suggesting that the primary cells were mainly VECs.

In order to simulate the hypoxic environment of PU, VECs were cultured in a hypoxic incubator and subjected to gene sequencing. The results showed that the gene expression of hypoxic VECs was significantly different from that of normoxic VECs (Additional file 1: Fig. S2A, B). Heat map analysis suggested that the expressions of ferroptosis-promoting genes *acsl4*, *ptgs2*, and *nox1* and autophagy genes *atg4b* and *atg7* were increased in VECs after hypoxia culture, whereas the expressions of anti-ferroptosis genes *fth1* and *slc7a3* were decreased (Additional file 1: Fig. S2C). The Kyoto Encyclopedia of Genes and Genomes (KEGG) enrichment analysis results for the upregulated genes suggested that the P53 signaling pathway was significantly enriched in VECs after hypoxia culture (Additional file 1: Fig. S2D). On the other hand, the Gene Ontology (GO) analysis results indicated that the epithelial cell proliferation, secretory vesicle, and exocytic vesicle pathways were significantly enhanced in VECs after normoxic culture as compared with those in VECs after hypoxia culture (Additional file 1: Fig. S2E).

### VEC-derived EVs carrying MFGE8 inhibited the autophagy-induced ferroptosis of VECs under hypoxia

**Fig. 2:**
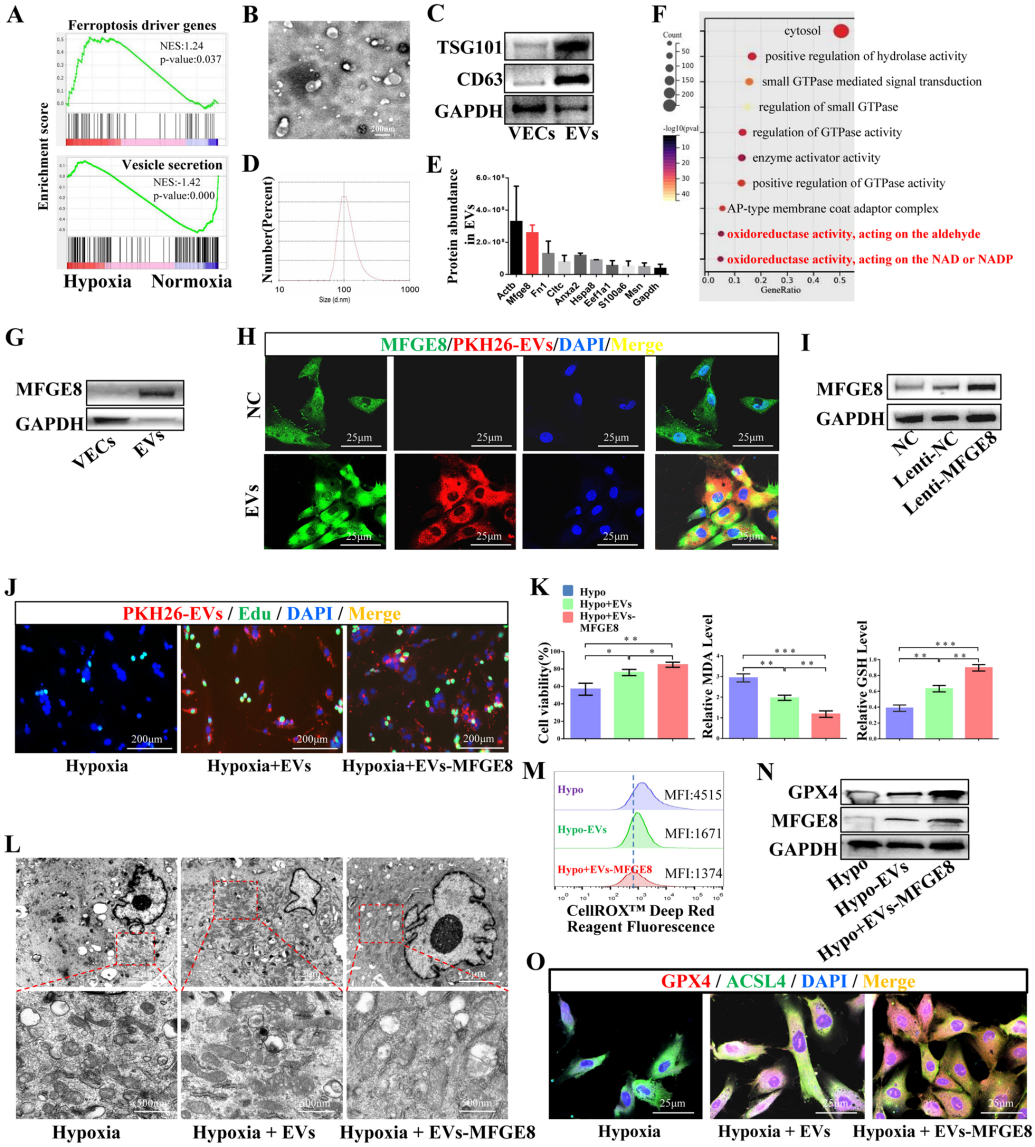
MFGE8-engineered EVs secreted by VECs overexpressing MFGE8 more effectively inhibited hypoxia-induced ferroptosis in VECs. **A** Gene Set Enrichment Analysis of activation pathways in VECs treated with or without hypoxia. **B**–**D** The morphology, specific protein, and particle size of EVs were assessed by transmission electron microscopy, western blotting, and a zeta particle size analyzer. **E** Abundance analysis of proteins in EVs by mass spectrometry. **F** GO enrichment analysis of activation pathways in EVs. **G** The MFGE8 expression in VECs and EVs was detected. **H** Representative immunofluorescence staining of EVs carrying MFGE8 into VECs. **I** Representative western blotting of MFGE8 in VECs with or without Lenti-MFGE8. **J**, **K** EdU staining, cell viability, MDA levels, and GSH levels in VECs treated with hypoxia, hypoxia + EVs, or hypoxia + EVs-MFGE8. The concentration of EVs or EVs-MFGE8 was 40 μg/ml. **L**, **M** Mitochondrial morphology and ROS levels characterized by the mean fluorescence intensity (MFI) were determined in VECs treated as above. **N**, **O** Representative western blotting assay of GPX4 and MFGE8 and immunofluorescence staining of GPX4 and ACSL4 in VECs treated with hypoxia, hypoxia + EVs, or hypoxia + EVs-MFGE8. NC: normal control. ns: *p* > 0.05; *p < 0.05; **p < 0.01; ***p < 0.001.

The Gene Set Enrichment Analysis (GSEA) of gene sequencing results for both normoxic and hypoxic VECs showed that the ferroptosis driver genes were significantly enriched in hypoxic VECs, whereas the vesicle secretion-related genes were significantly enriched in normoxic VECs (Fig. 2A). Based on this, we investigated the role of EVs secreted by normoxic VECs in regulating the ferroptosis of VECs under hypoxia and simulated the role of EVs secreted by normal VECs near PU in regulating the ferroptosis of VECs under hypoxia in PU. VECs were cultured under normoxia, and VEC-derived EVs were isolated. EVs, which were identified via TEM, western blotting, and particle size detection (Fig. 2B–D), were highly expressed in CD63 and TSG101, with the average particle size being approximately 100 nm. The death of VECs increased after hypoxic culture; in contrast, the death of VECs under hypoxia was inhibited by EVs secreted by normoxic VECs (Additional file 1: Fig. S3A). Moreover, the results for iron, MDA, and GSH levels and for mitochondrial changes related to ferroptosis indicated that hypoxia promoted an increase in iron and MDA levels and enhanced the mitochondrial shrinkage and collapse in the ferroptosis of VECs, whereas EVs effectively reversed the changes associated with hypoxia-induced ferroptosis (Additional file 1: Fig. S3B–C). To further clarify the regulatory role of EVs in ferroptosis under hypoxia, we examined the expression levels of the ferroptosis-related protein GPX4 and the mean fluorescence intensity (MFI) associated with ROS levels. Hypoxia led to a decreased GPX4 expression and an increased ROS expression, whereas EVs increased the GPX4 expression and inhibited the hypoxia-induced ROS levels (Additional file 1: Fig. S3D–E). EVs contained various components such as proteins, metabolites, and nucleic acids [36, 37]. We performed mass spectrometric analysis of EVs to identify the components of EVs that perform anti-ferroptosis function. Protein abundance analysis suggested that MFGE8 was abundant in EVs (Fig. 2E). GO enrichment analysis of proteins in EVs showed that the EVs had oxidoreductase activity that acted on aldehyde, NAD, and NADP (Fig. 2F). Additionally, we used the western blotting assay to further confirm whether the abundance of MFGE8 in EVs increased as compared with that in VECs, and the results showed that the MFGE8 expression levels were higher in EVs than in VECs (Fig. 2G). Moreover, EVs could carry MFGE8 into the VECs (Fig. 2H). We overexpressed MFGE8 in VECs with MFGE8 lentivirus (Lenti-MFGE8) to construct MFGE8-engineered EVs (EVs-MFGE8), and we verified that the MFGE8 expression levels in VECs transfected with Lenti-MFGE8 (Lenti-MFGE8-VECs) were higher than those in VECs and Lenti-NC-VECs (Fig. 2I). EVs secreted by VECs or EVs-MFGE8 secreted by Lenti-MFGE8-VECs were extracted. EVs and EVs-MFGE8 were used to inhibit ferroptosis in hypoxic VECs. The EdU staining results indicated that EVs-MFGE8 more effectively promoted the proliferation of hypoxic VECs than EVs (Fig. 2J). Cell viability and GSH levels increased, whereas MDA levels decreased in VECs treated with hypoxia + EVs-MFGE8 compared with those in VECs treated with hypoxia or hypoxia + EVs (Fig. 2K). Moreover, mitochondrial shrinkage and cleavage, including ferroptosis-related ROS levels, were obviously inhibited in VECs treated with hypoxia + EVs-MFGE8 (Fig. 2L–M). Thus, EVs-MFGE8 more effectively inhibited ferroptosis than EVs. The expressions of MFGE8 and GPX4 were analyzed using the western blotting assay and immunofluorescence staining, which indicated that EVs-MFGE8 could carry more MFGE8 into VECs than EVs, thus inhibiting ferroptosis caused by hypoxia more effectively (Fig. 2N, O).

Subsequently, the effect and mechanism of MFGE8 on inhibiting ferroptosis were verified. The KEGG enrichment analysis of all differential genes in hypoxic and normoxic VECs showed that the P53 signaling pathway associated with ferroptosis and the lysosome pathway associated with autophagy were significantly activated (Additional file 1: Fig. S4A). To verify whether autophagy promoted ferroptosis, erastin and 3-methyladenine were used to induce ferroptosis and inhibit autophagy, respectively. It was concluded that autophagy promoted ferroptosis and that the inhibition of autophagy also diminished the ferroptosis of VECs (Additional file 1: Fig. S4B). Subsequently, we examined lipid peroxidation, iron, MDA, and GSH levels, as well as mitochondrial changes. The results indicated that erastin-induced ferroptosis was inhibited by the inhibition of autophagy (Additional file 1: Fig. S4C–E). Correlation analysis showed that MFGE8 was negatively correlated with the ferroptosis genes *ptgs2*, *nox1*, and *acsl4*, as well as with the autophagy genes *atg4b* and *atg7*; furthermore, the ferroptosis genes *ptgs2*, *nox1*, and *acsl4* were significantly positively correlated with the autophagy genes *atg4b* and *atg7* (Additional file 1: Fig. S5A). Based on the above results for the correlation of MFGE8 with ferroptosis and autophagy, we overexpressed MFGE8 in VECs. Erastin increased the expressions of the proteins P53, ACSL4, and LC3B/A, which promoted ferroptosis, whereas MFGE8 increased the expressions of the proteins GPX4, which alleviated ferroptosis (Additional file 1: Fig. S5B). The MFI was used to detect the ROS levels, the transwell was used to detect the invasive function of VECs, and immunofluorescence was used to identify the role of MFGE8 in regulating the expression of the proteins LC3B, GPX4, and ACSL4. The results revealed that MFGE8 inhibited autophagy and alleviated the ferroptosis of VECs (Additional file 1: Fig. S5C–F). Thus, VEC-derived EVs played an important role in inhibiting the occurrence of ferroptosis by carrying MFGE8 into VECs to exercise inhibition of autophagy-induced ferroptosis.

### CD13 expression increased, whereas MFGE8 expression decreased in PU skin tissues

**Fig. 3:**
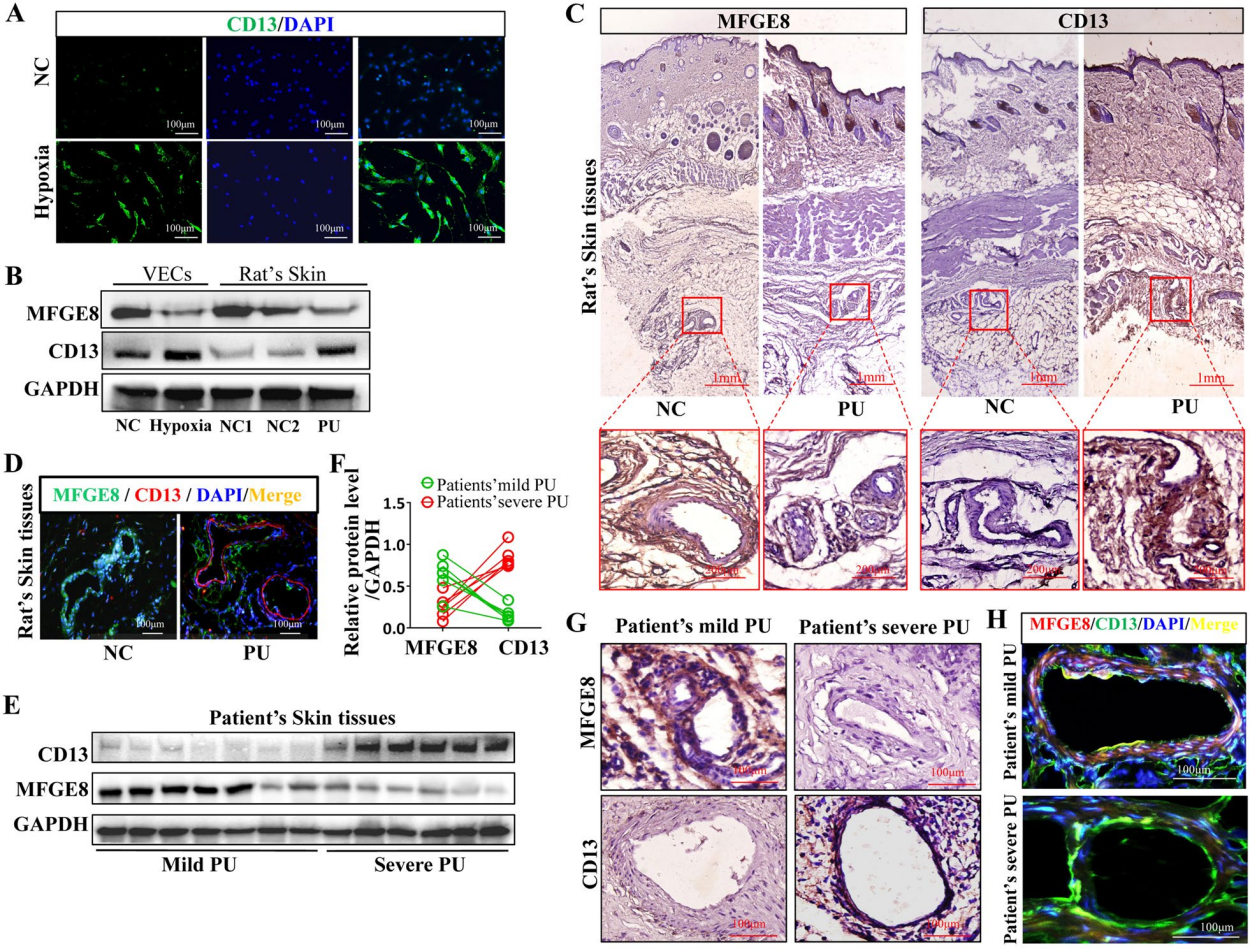
CD13 expression increased and MFGE8 expression decreased in PU tissues. **A**, **B** CD13 and MFGE8 expressions in VECs under normoxia and hypoxia or in rats’ skin tissues with or without PU were detected by immunofluorescence and western blotting. **C**, **D** CD13 and MFGE8 expressions in rats’ skin tissues with or without PU were detected by immunofluorescence and immunohistochemistry. **E**–**H** The expression levels of CD13 and MFGE8 in mild and severe PU tissues were detected by western blotting and quantitative analysis of expression levels, immunofluorescence, and immunohistochemistry. NC: normal control

CD13 and MFGE8 expressions were detected in VECs and PU tissues to analyze the role of CD13 and MFGE8 in the occurrence and development of PU. Immunofluorescence and western blotting results indicated that the CD13 expression increased, whereas the MFGE8 expression decreased in VECs treated with hypoxia and PU skin tissues in rats as compared with the expression of CD13 and MFGE8 in VECs treated with normoxia or normal skin tissues in rats (Fig. 3A, B). Immunohistochemical and immunofluorescence staining of rats’ normal and PU skin tissues showed that the CD13 expression was increased in VECs in PU skin tissues, whereas the MFGE8 expression was decreased in VECs in PU skin tissues (Fig. 3C, D). Subsequently, we collected mild and severe PU tissues from patients and then performed western blotting and relative protein expression analysis, immunohistochemistry, and immunofluorescence staining (Fig. 3E–H). The CD13 expression was increased in skin tissues or VECs in severe PU, whereas the MFGE8 expression was decreased in skin tissues or VECs in severe PU as compared with that observed in skin tissues in mild PU.

### NPs carried the encapsulated purified MFGE8 protein into VECs

**Fig. 4:**
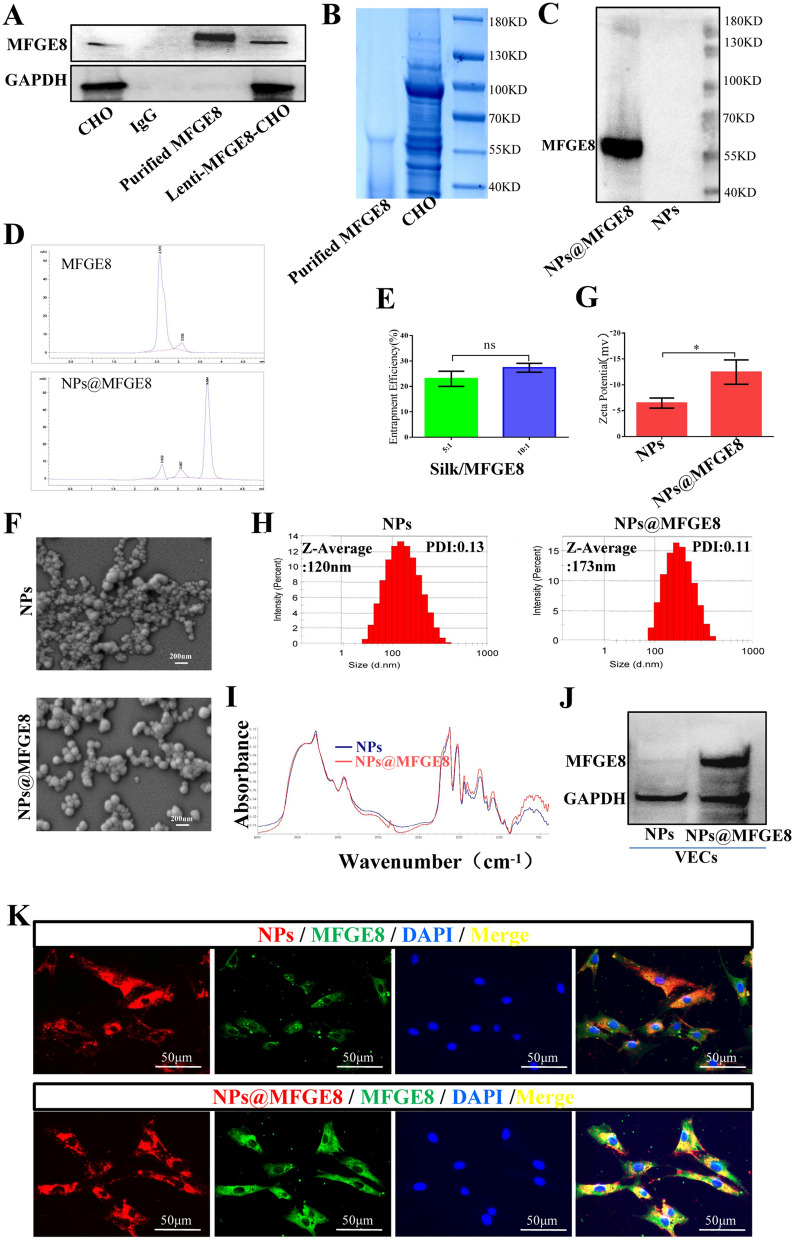
MFGE8-coated NPs (NPs@MFGE8) could carry MFGE8 produced by CHO cells into VECs. **A** Expression of MFGE8 in CHO cells, IgG, purified MFGE8, and Lenti-MFGE8-CHO cells in rats was analyzed by western blotting. **B** The purified MFGE8 protein was detected by Coomassie bright blue staining. **C** Representative western blotting of MFGE8 in NPs coated with or without MFGE8. **D** HPLC of purified MFGE8 and NPs@MFGE8 proteins lysed with lithium bromide (LiBr) (9.3 mol/L). The peaks of the MFGE8 protein were 2.570 min and 2.632 min, respectively. **E** The encapsulation rate of NPs@MFGE8 at a silk fibroin-to-MFGE8 ratio of 5:1 and 10:1 was determined via HPLC. **F**–**H** The morphology, zeta potential, polydispersity index (PDI) and particle size of NPs and NPs@MFGE8 were analyzed via scanning electron microscopy (SEM) and using a zeta potential and particle size analyzer. **I** Fourier transform infrared spectrometry was conducted for NPs and NPs@MFGE8. **J**, **K** Representative western blotting analysis and immunofluorescence staining of MFGE8 in VECs treated with NPs or NPs@MFGE8. NC: normal control. ns: *p* > 0.05; *p < 0.05; **p < 0.01; ***p < 0.001

CHO cells are the most common host used for the production of purified protein [38]. In this study, CHO cells were also used to prepare and extract the purified MFGE8 protein. Western blotting and Coomassie blue staining were conducted to detect the MFGE8 expression in CHO, IgG polypeptide, purified MFGE8, and Lenti-MFGE8-CHO. The results showed that purified MFGE8 could be effectively obtained using the magnetic bead method (Fig. 4A, B). MFGE8 was then encapsulated in silk fibroin NPs to simulate the role of VEC-derived EVs in disease therapy. Compared with NPs, the MFGE8 protein could be detected in NPs@MFGE8 (Fig. 4C). HPLC was performed to determine the obtained purified MFGE8 proteins and the NPs@MFGE8 encapsulation rate at a silk fibroin-to-MFGE8 ratio of 5:1 and 10:1. After the purified MFGE8 and NPs@MFGE8 proteins were dissolved by LiBr (9.3 mol/L), their waveforms were detected, and the peaks of the MFGE8 protein were 2.570 min and 2.632 min, respectively (Fig. 4D). The waveform of the purified MFGE8 protein was unique and similar to that of the MFGE8 in NPs@MFGE8. Moreover, the encapsulation rates were approximately 23% and 27%, respectively, albeit without statistical difference (Fig. 4E). The characteristics of MFGE8 released from NPs@MFGE8 were also detected by HPLC. On the third day, about 15% of MFGE8 in NPs@MFGE8 was released, and about 35% was released by the seventh day in vitro (Additional file 1: Fig. S6). The morphology, zeta potential, and particle size of NPs and NPs@MFGE8 were examined via SEM and using a zeta potential and particle size analyzer. NPs and NPs@MFGE8 were spherical particles, with a zeta potential of approximately − 7 mV and − 12 mV, respectively, an average particle size of approximately 120 nm and 173 nm, respectively, and a polydispersity index (PDI) of 0.13 and 0.11, respectively (Fig. 4F–H). FT-IR indicated a significant difference between NPs and NPs@MFGE8 at the 2200–2400 cm^−1^ band (Fig. 4I). RBITC-NPs and RBITC-NPs@MFGE8 were co-cultured with VECs. Western blotting and immunofluorescence results confirmed that NPs@MFGE8 could carry the MFGE8 protein into VECs as compared with NPs (Fig. 4J–K). The clearance of NPs after uptake by VECs was analyzed based on the mean fluorescence intensity (MFI). The MFI of VECs was gradually decreased from day 1 to day 7 (Additional file 1: Fig. S7A, B), but the clearance rate increased gradually and was approximately 70% on the seventh day, which was independent of whether the NPs are coated or not with MFGE8 (Additional file 1: Fig. S7C). The above results show that silk fibroin NPs@MFGE8 could simulate EVs carrying MFGE8.

### NPs@MFGE8 simulated EVs-MFGE8 to inhibit mitochondrial autophagy and alleviate ferroptosis

**Fig. 5:**
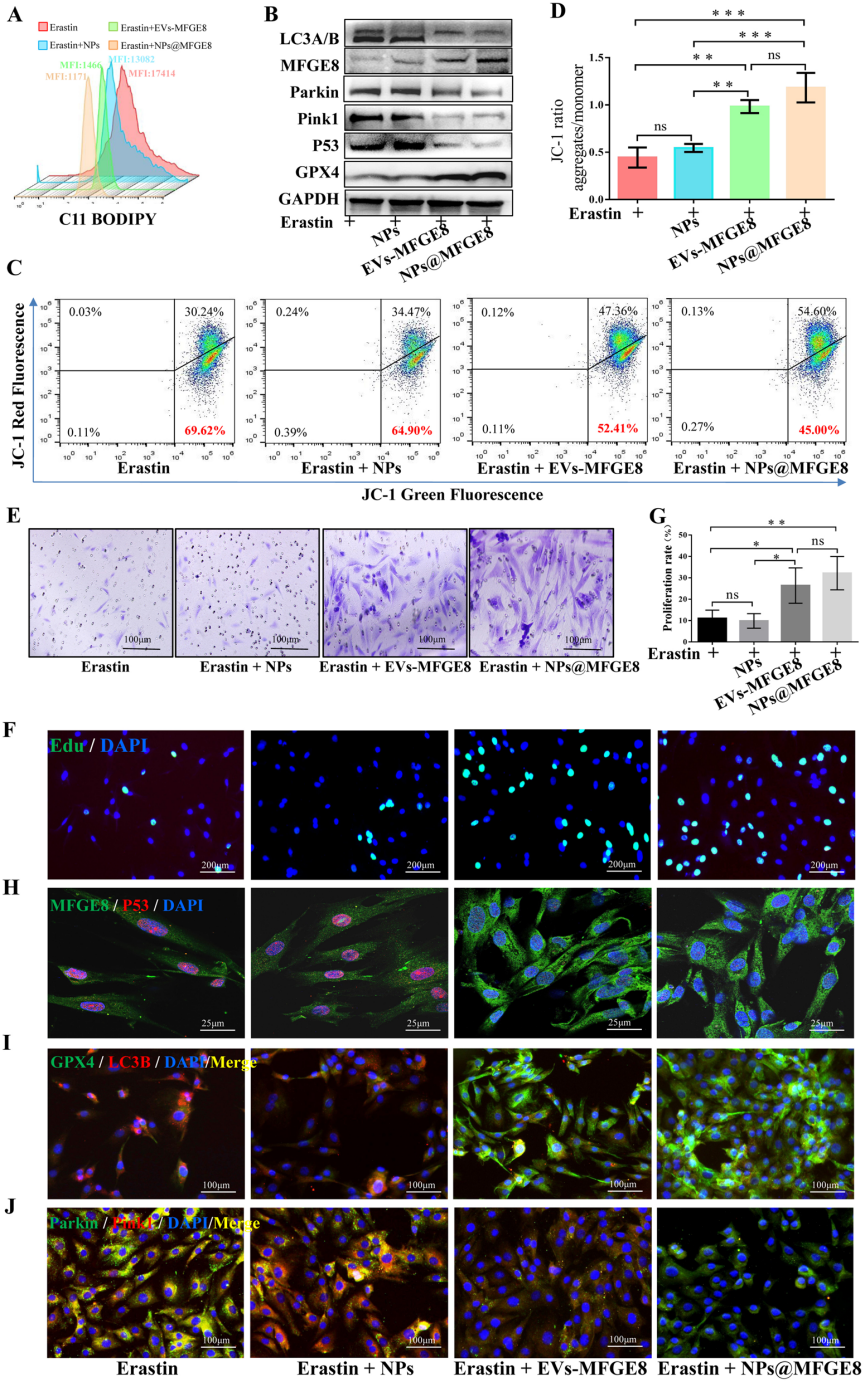
NPs@MFGE8 simulated EVs-MFGE8 effectively to inhibit mitochondrial autophagy-induced ferroptosis. **A** Lipid peroxidation levels of VECs treated with erastin, erastin + NPs, erastin + EVs-MFGE8, and erastin + NPs@MFGE8. The concentration of EVs-MFGE8 and NPs@MFGE8 was 40 μg/ml and 500 μg/ml, respectively. **B** Western blotting of LC3A/B, MFGE8, parkin, pink1, P53, and GPX4 in VECs treated as above. **C**, **D** The mitochondrial membrane potential was detected by flow cytometry, and statistical analysis of the aggregate-to-monomer ratio was performed. **E**–**G** Transwell assay, EdU staining, and statistical analysis of proliferation rate were used to detect the invasive and proliferative abilities of VECs treated as above. **H**–**J** Representative immunofluorescence staining of MFGE8/P53, GPX4/LC3B, and parkin/pink1 in VECs treated with erastin, erastin + NPs, erastin + EVs-MFGE8, and erastin + NPs@MFGE8. NC: normal control. ns: *p* > 0.05; *p < 0.05; **p < 0.01; ***p < 0.001

Whether NPs@MFGE8 and EVs-MFGE8 had similar functions in inhibiting autophagy-induced ferroptosis has not been clear. First, the critical concentration of NPs@MFGE8 in vitro experiments was detected, and the CCK-8 assay showed that the critical concentration for enhancing VECs viability might be 500 μg/ml (Additional file 1: Fig. S8). The dosage of MFGE8 in NPs@MFGE8 (500 μg/ml) was determined by HPLC. The peaks of MFGE8 in the pured MFGE8 protein and NPs@MFGE8 were 2.053 min and 2.127 min, respectively (Additional file 1: Fig. S9A, B). The concentration of MFGE8 might be about 10 μg/ml (Additional file 1: Fig. S9C). Then, VECs were treated with erastin, erastin + NPs, erastin + EVs-MFGE8, and erastin + NPs@MFGE8 to evaluate the effect of NPs@MFGE8 emulation of EVs-MFGE8 on the inhibition of ferroptosis. Based on the MFI value, the erastin-induced increase in lipid peroxidation levels was determined to be decreased by NPs@MFGE8 and EVs-MFGE8 (Fig. 5A). Afterwards, western blotting was performed to detect the expression of the proteins LC3B/A, MFGE8, parkin, pink1, P53, and GPX4 (Fig. 5B). Erastin induced an increase in LC3B/A, parkin, pink1, and P53 in VECs, whereas NPs@MFGE8 and EVs-MFGE8 inhibited the increase in these ferroptosis-related proteins. Flow cytometry and statistical analysis of the aggregate-to-monomer ratio also showed that NPs@MFGE8 and EVs-MFGE8 could increase the aggregate-to-monomer ratio (Fig. 5C, D). The transwell results indicated that both NPs@MFGE8 and EVs-MFGE8 effectively promoted the invasive function of VECs (Fig. 5E). EdU staining and statistical analysis results for the proliferation rate showed that both NPs@MFGE8 and EVs-MFGE8 effectively promoted the proliferation of erastin-treated VECs (Fig. 5F–G). NPs, EVs-MFGE8, or NPs@MFGE8 were co-cultured with erastin-treated VECs, and immunofluorescence staining of MFGE8/P53 (Fig. 5H), GPX4/LC3B (Fig. 5I), and parkin/pink1 (Fig. 5J) was then performed. NPs@MFGE8 and EVs-MFGE8 reduced the erastin-induced expression of P53, LC3B, parkin, and pink1 in VECs. Therefore, NPs@MFGE8 could effectively simulate EVs-MFGE8 to inhibit mitochondrial autophagy and alleviate ferroptosis.

### Cross-linked NGR NPs@MFGE8 targeted CD13 of VECs under hypoxia

**Fig. 6:**
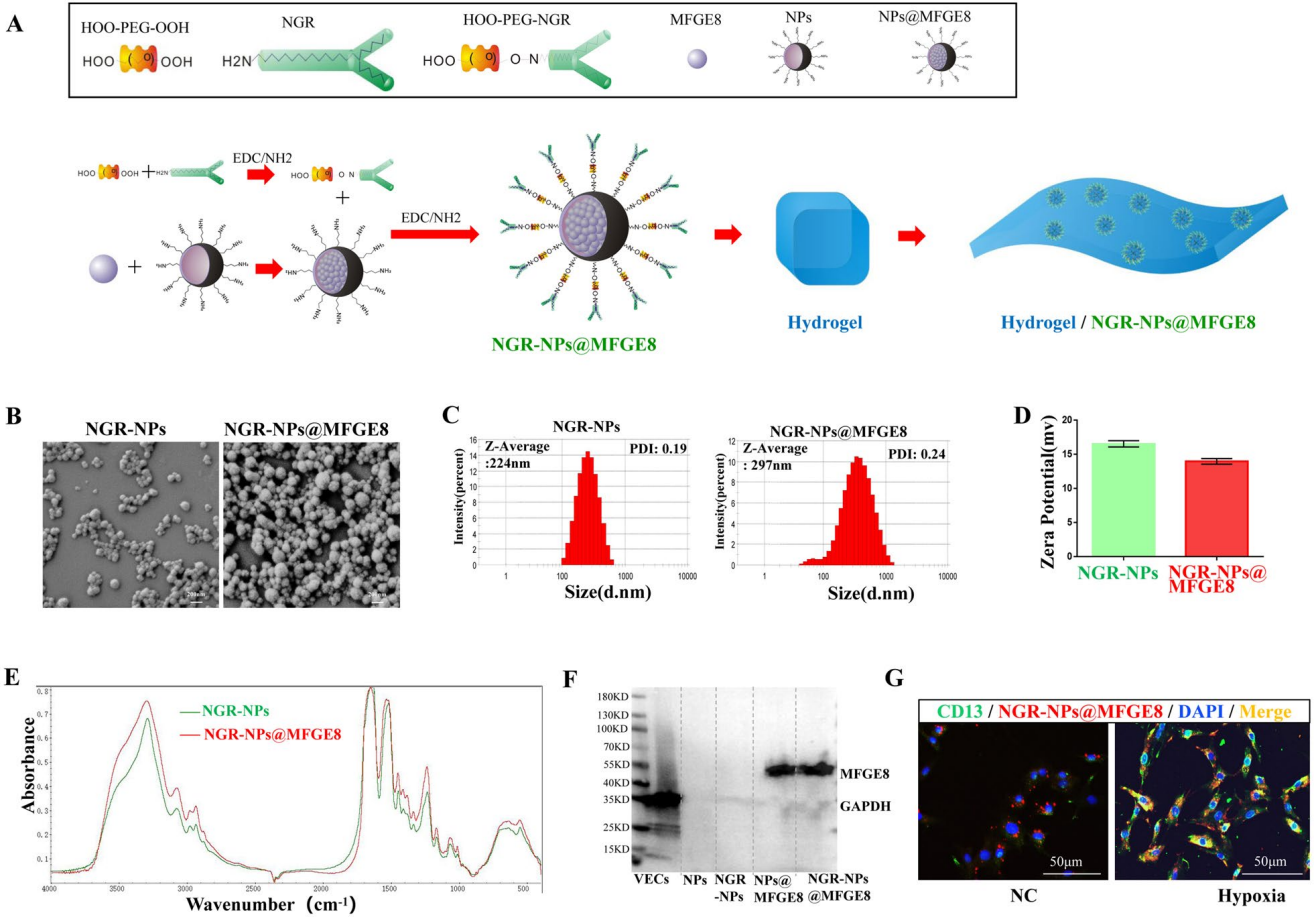
Cross-linked NGR NPs@MFGE8 (NGR-NPs@MFGE8) carried MFGE8 into VECs via targeting CD13 under hypoxia. **A** Flow chart of the synthesis of NGR-NPs@MFGE8. **B**–**D** The morphology, particle size, PDI and zeta potential of NGR-NPs and NGR-NPs@MFGE8 were examined via SEM and using a zeta potential and particle size analyzer. **E** Fourier transform infrared spectrometry was conducted for NGR-NPs and NGR-NPs@MFGE8. **F** The expression of MFGE8 in VECs, NPs, NGR-NPs, NPs@MFGE8, and NGR-NPs@MFGE8 was detected by western blotting. **G** Uptake of NGR-NPs@MFGE8 by VECs treated with or without hypoxia was determined by immunofluorescence

For the construction of cross-linked NGR NPs coated with MFGE8 (NGR-NPs@MFGE8), HOOC-PEG-COOH was used as a bridge to cross-link the NGR peptide and silk fibroin NPs. The flow chart illustrating the construction of NGR-NPs@MFGE8 is presented in Fig. 6A. The morphology, particle size, and potential of NGR-NPs and NGR-NPs@MFGE8 were then examined via SEM and using a zeta potential and particle size analyzer. NGR-NPs and NGR-NPs@MFGE8 were spherical particles, with an average particle size of approximately 224 nm and 297 nm and PDI of 0.19 and 0.24, respectively (Fig. 6B, C), and a zeta potential of approximately 16.5 mV and 14 mV, respectively (Fig. 6D). FT-IR indicated a significant difference between NGR-NPs and NGR-NPs@MFGE8 at the 3400–3600 cm^−1^ band (Fig. 6E). We also investigated whether NGR-NPs and NGR-NPs@MFGE8 could effectively carry MFGE8 into hypoxic-treated VECs. Western blotting results showed that NGR-NPs@MFGE8 and NPs@MFGE8 could carry more MFGE8 than VECs, NPs, and NRG-NPs (Fig. 6F). Moreover, immunofluorescence results indicated that hypoxic-treated VECs expressed more CD13 and could be more rapidly targeted by NGR-NPs@MFGE8 than normal VECs (Fig. 6G).

### Characterization of hydrogel sustained-release carrier

**Fig. 7:**
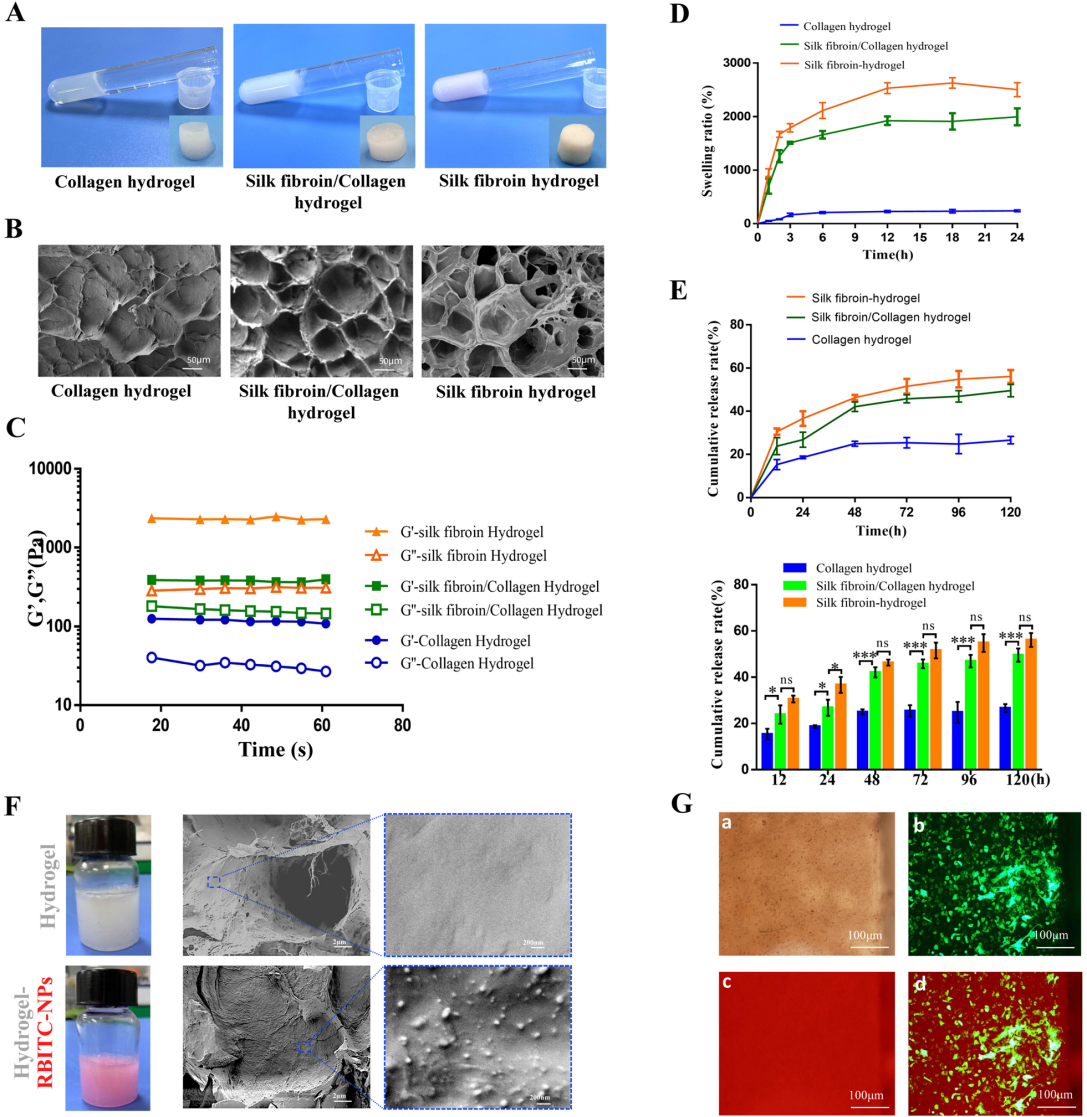
Characterization of hydrogel sustained-release carrier. **A**, **B** Morphological and structural characteristics of the collagen hydrogel, silk fibroin/collagen hydrogel, and silk fibroin hydrogel. **C** The G' and G'' of the three aforementioned hydrogels. **D** Swelling ratios of the collagen hydrogel, silk fibroin/collagen hydrogel, and silk fibroin hydrogel. **E** NPs release rates and statistical analysis of the collagen hydrogel, silk fibroin/collagen hydrogel, and silk fibroin hydrogel. **F** Preparation of silk fibroin/collagen hydrogel (white) and silk fibroin/collagen hydrogel coated with RBITC-labeled NPs (RBITC-NPs) (red), and the distribution of NPs in hydrogel was observed by SEM. **G** Culture of VECs on the Silk fibroin/Collagen hydrogel. a. The morphology of cells on the Silk fibroin/Collagen hydrogel under white light. b. The morphology of cells transfected with Lenti-NC-Green on the Silk fibroin/Collagen hydrogel. c. Silk fibroin/Collagen hydrogel was stained with RBITC. d. The morphology of the merged cells on the Silk fibroin/Collagen hydrogel. ns: *p* > 0.05; *p < 0.05; **p < 0.01; ***p < 0.001

To construct the sustained-release carrier of NPs, we prepared collagen hydrogel, silk fibroin/collagen hydrogel, and silk fibroin hydrogel (Fig. 7A). The structure of these three hydrogels was loose and porous, as observed on SEM (Fig. 7B). By introducing collagen components, the G' and G'' of silk fibroin/collagen hydrogel were shown to be lower than those of silk fibroin hydrogel but higher than those of collagen hydrogel (Fig. 7C). The swelling ratio of silk fibroin/collagen hydrogel was shown to be lower than that of silk fibroin hydrogel but higher than that of collagen hydrogel (Fig. 7D). The sustained-release carrier of NPs was constructed by mixing the NPs with hydrogel. Although the NP release rate of silk fibroin/collagen sustained-release carrier was lower than that of silk fibroin hydrogels and higher than that of collagen hydrogels over time (Fig. 7E), the cumulative release rate of silk fibroin/collagen hydrogel was not significantly different from that of silk fibroin hydrogel after 48 h. The silk fibroin/collagen hydrogel carriers were gradually degraded, and approximately 20% of the hydrogel carriers were degraded at day 10 (Additional file 1: Fig. S10). Therefore, in the treatment of skin PU, silk fibroin/collagen hydrogel was used as the sustained-release carrier of NPs to achieve a better therapeutic effect. Compared with the silk fibroin/collagen hydrogel, the silk fibroin/collagen hydrogel loaded with RBITC-NPs appeared red, and the morphology of NPs in the hydrogel could be observed on SEM (Fig. 7F). Subsequently, the VECs were cultured on the silk fibroin/collagen hydrogel to detect biocompatibility. After 2 d, the cells grew well on the hydrogel and were spindle-shaped (Fig. 7G).

### Cross-linked NGR-NPs@MFGE8 targeted VECs in PU tissues

**Fig. 8:**
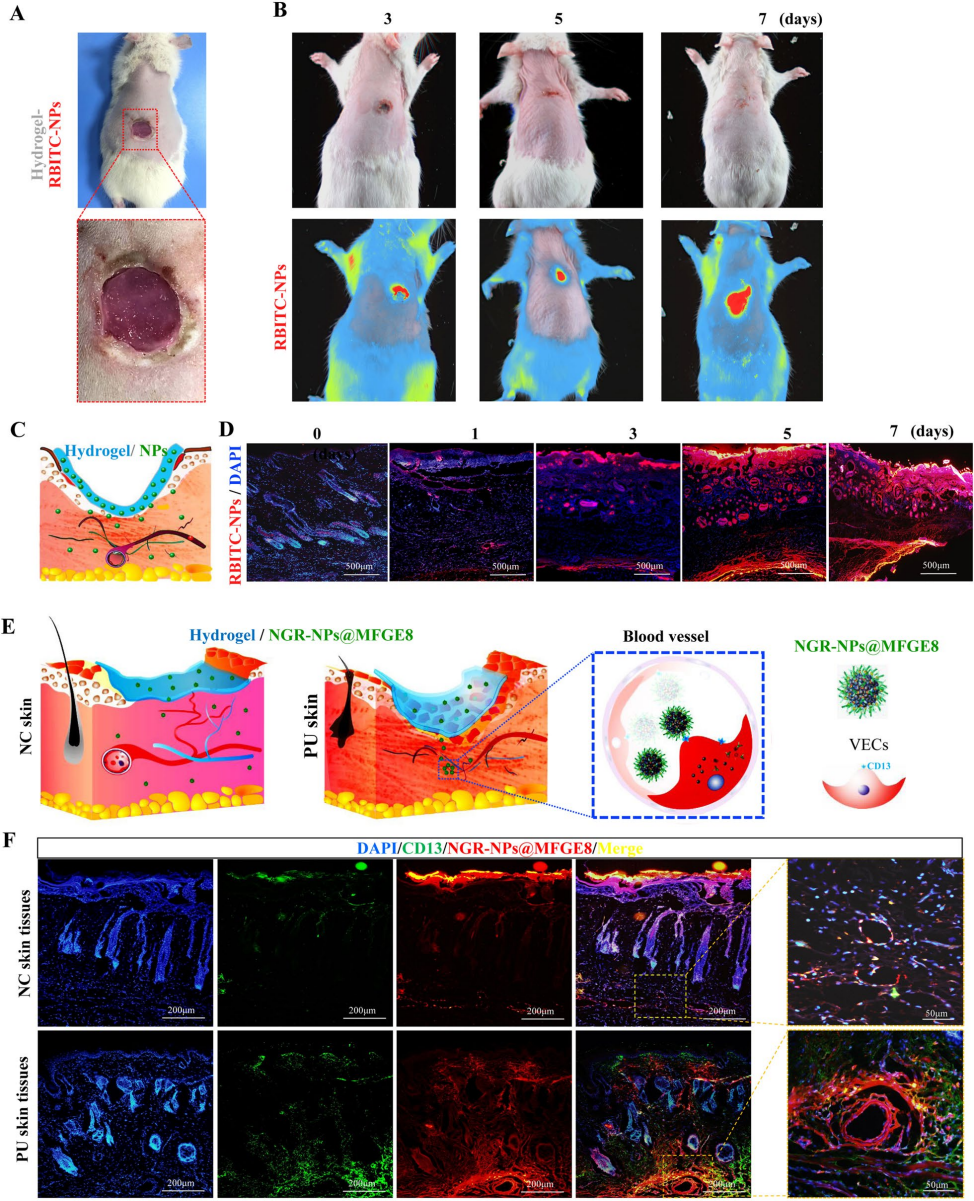
The silk fibroin/collagen hydrogel sustained-release carrier released NGR-NPs@MFGE8 targeting VECs in PU tissue. **A** The hydrogel loaded with RBITC-NPs was applied to the ulcer surface and continued by changing the hydrogel daily for 5 d. **B** In vivo imaging was performed on the skin pressure ulcer at days 3, 5, and 7. **C** The diagram of the hydrogel sustained-release carrier continuously releasing NPs into the surrounding tissue on the PU tissue. **D** Fluorescence observation was used to detect the diffusion of RBITC-NPs in skin tissues at days 0, 1, 3, 5, and 7. **E** The diagram of the hydrogel sustained-release carrier continuously releasing NGR-NPs@MFGE8 into normal or PU tissues. **F** Fluorescence observation was used to analyze the targeting effect of RBITC-NGR-NPs@MFGE8 on blood vessels. NC: normal control

After debriding the necrotic tissues in PU, the silk fibroin/collagen hydrogel loaded with RBITC-NPs was applied to PU (Fig. 8A). The treatment was continued by changing the hydrogel daily for 5 d. Subsequently, in vivo imaging was performed on skin PU at days 3, 5, and 7. With the extension of hydrogel treatment time, the RBITC-NPs could exist in the skin for a long period (Fig. 8B). As shown in the pattern diagram, NPs were continuously released by the hydrogel sustained-release carrier and diffusely spread into the surrounding area in PU tissues (Fig. 8C). Fluorescence observation of PU tissues at days 0, 1, 3, 5, and 7 revealed that RBITC-NPs considerably spread in the skin with the extension of time (Fig. 8D). To evaluate whether RBITC-NGR-NPs@MFGE8 could target VECs in PU tissues, the NC group was not damaged with PU; however, the surface skin structure was partially removed using a blade. Then the skin in the NC and PU groups were treated with RBITC-NGR-NPs@MFGE8-loaded hydrogel. As could be seen from the diagram, the NGR-NPs@MFGE8 released by the silk fibroin/collagen hydrogel spread around the normal skin tissues. The NGR-NPs@MFGE8 released by the silk fibroin/collagen hydrogel specifically targeted CD13, which was highly expressed in VECs due to hypoxia in PU tissues but had no specific targeting effect on VECs that did not express CD13 in NC skin tissues (Fig. 8E). Fluorescence observation also indicated that the blood vessels in the PU group could be effectively targeted by RBITC-NGR-NPs@MFGE8, as compared with those in the NC skin tissues (Fig. 8F).

### NGR-NPs@MFGE8 inhibited mitochondrial autophagy-induced ferroptosis and promoted wound healing in PU

**Fig. 9:**
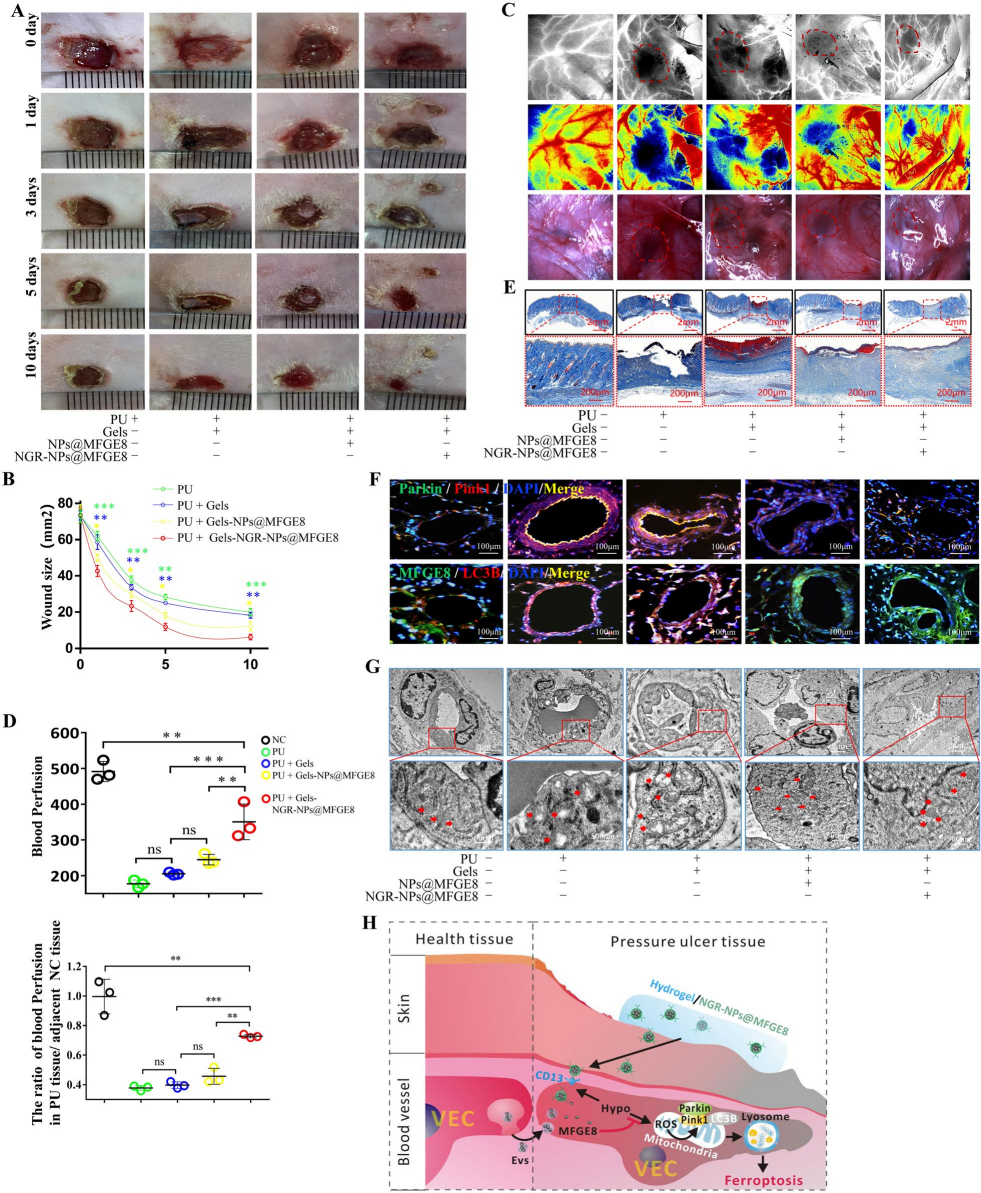
The hydrogel sustained-release carrier inhibited mitochondrial autophagy-induced ferroptosis by releasing NGR-NPs@MFGE8 and promoted rapid PU healing. **A**, **B** Wound size and its statistical analysis at days 0, 1, 3, 5, and 10. *(green): PU *vs* PU + Gels-NGR-NPs@MFGE8, *(blue): PU + Gels *vs* PU + Gels-NGR-NPs@MFGE8, *(yellow): PU + Gels-NPs@MFGE8 *vs* PU + Gels-NGR-NPs@MFGE8. C, D The blood perfusion volume and the blood perfusion ratio between the PU tissues and PU-adjacent normal tissues were determined using the RWD Laser Speckle Imaging System in the NC group, PU group, PU + Gels group, PU + Gels-NPs@MFGE8 group, and PU + Gels-NGR-NPs@MFGE8 group. (E, F) Masson staining and immunofluorescence staining of parkin/pink1 and MFGE8/LC3B were conducted to detect wound healing and ferroptosis of VECs in these five groups. (G) Mitochondrial changes in VECs were observed using transmission electron microscopy. (H) Illustration of the postulated mechanism by which Gels-NGR-NPs@MFGE8 targeted the CD31 to inhibit the ferroptosis of VECs and accelerate wound healing. NC: normal control. ns: *p* > 0.05; *p < 0.05; **p < 0.01; ***p < 0.001

To determine the effect of NGR-NPs@MFGE8 on PU healing, we evenly divided 15 rats into five groups—namely, the NC (*n* = 3), PU (*n* = 3), PU + silk fibroin/collagen hydrogel (Gels) (*n* = 3), PU + silk fibroin/collagen hydrogel-NPs@MFGE8 (Gels-NPs@MFGE8) (*n* = 3), and PU + silk fibroin/collagen hydrogel-NGR-NPs@MFGE8 (Gels-NGR-NPs@MFGE8) (*n* = 3) groups. Statistical analysis results for wound healing (including the wound size) at days 0, 1, 3, 5, and 10 indicated that NGR-NPs@MFGE8 had the most obvious effect on wound healing (Fig. 9A, B). The blood perfusion volume and the blood perfusion ratio between the PU tissues and PU-adjacent normal tissues were detected using the RWD Laser Speckle Imaging System. The results indicated that NGR-NPs@MFGE8 showed the most obvious effect with respect to the promotion of blood perfusion recovery in wounds (Fig. 9C, D). Masson staining also suggested that NGR-NPs@MFGE8 had the most obvious effect on the promotion of collagen deposition and wound healing (Fig. 9E). Moreover, an additional nine rats were used to analyze the effect of Gels-NPs on promoting PU healing. Compared with the Gels group (*n* = 3), Gels-NPs (*n* = 3) showed no significant difference in promoting ulcer healing, but Gels-NPs@MFGE8 (*n* = 3) significantly accelerated the ulcer healing after 6 days than Gels-NPs (Additional file 1: Fig. S11 A–B). The results of masson staining also showed that Gels-NPs had no significant difference from Gels in promoting collagen deposition, but Gels-NPs was significantly lower than Gels-NPs@MFGE8 (Additional file 1: Fig. S11C). Immunofluorescence staining was performed on parkin, pink1, MFGE8, and LC3B in VECs (Fig. 9F), whereas immunohistochemistry was conducted on parkin, pink1, MFGE8, and GPX4 in VECs (Additional file 1: Fig. S12). Compared with those in VECs from the normal skin, the expressions of parkin, pink1, and LC3B in VECs in PU tissues were significantly increased, whereas the expressions of MFGE8 and GPX4 were decreased. After treatment, Gels-NGR-NPs@MFGE8 was found to significantly increase the expressions of MFGE8 and GPX4 but decrease the expressions of parkin and pink1 in VECs in PU tissues as compared with Gels and Gels-NPs@MFGE8. Moreover, mitochondrial TEM showed that Gels-NGR-NPs@MFGE8 was the most effective in suppressing mitochondrial swelling in VECs in PU tissues (Fig. 9G). Although the NGR-NPs@MFGE8 in this study targeted VECs, they also had effects on fibroblasts and macrophages. The immunohistochemical staining of CD14 and α-SMA and statistical analysis showed that the number of macrophages in the PU group and PU + Gels groups was significantly increased (Additional file 1: Fig. 13SA, B), while the number of fibroblasts in the Gels-NPs@MFGE8 group and PU + Gels-NGR-NPs@MFGE8 group was significantly increased, especially in the PU + Gels-NGR-NPs@MFGE8 group (Additional file 1: Fig. 13SC, D). Therefore, MFGE8 might inhibit the infiltration of macrophages and inflammatory response in the ulcer site and promote the proliferation of fibroblasts and wound repair in the process of PU repair. Thus, as shown in Fig. 9H, our study aimed to elucidate the etiology and mechanism of PU and to develop a promising avenue for the promotion of PU healing by simulating EVs secreted by VECs. This was achieved by utilizing EV-derived NGR-NPs@MFGE8 encapsulated in the silk fibroin/collagen hydrogel to locally supplement and deliver MFGE8 into VECs via CD13 targeting specifically under hypoxia. In VECs in a hypoxic environment, MFGE8 could inhibit the increase in ROS levels and mitochondrial autophagy-induced ferroptosis, leading to the inhibition of inflammatory response, alleviation of blood vessel injury, promotion of collagen deposition and acceleration of skin PU healing.

## Discussion

PU remains a common disease globally, and its frequent recurrence makes it difficult to cure, thereby seriously threatening the health of patients and increasing the economic burden on society [39]. Only a few effective treatments are available in the clinic, and the mechanism underlying hypoxia-induced PU also remains unclear. Based on the CD13 protein enriched on the membrane of hypoxic VECs, we constructed NGR-labeled NPs carrying MFGE8 (NGR-NPs@MFGE8) to target VECs in PU tissues. By mixing with NGR-NPs@MFGE8, silk fibroin/collagen sustained-release carrier could release NGR-NPs@MFGE8 continuously to inhibit mitochondrial autophagy-induced ferroptosis, thus relieving the ulcers and promoting wound healing.

Regional hypoxia caused by repeated ischemia–reperfusion leads to PU, which are difficult to heal [6]. Hypoxia is also the main inducing factor for ferroptosis in various diseases [40–42]. Therefore, in the present study, we investigated whether ferroptosis caused by hypoxia occurred in PU tissues. We found that the ferroptosis level increased in PU tissues in patients and rats. Considering that alleviating the damage to VECs was beneficial to skin wound healing [43], we investigated whether ferroptosis, which might delay wound healing, occurred in VECs in PU tissues. The results indicated that ferroptosis occurred in VECs in hypoxic PU tissues, which further confirmed that the ferroptosis of VECs in PU tissues might be mainly caused by hypoxia. Although some studies on tumors have shown that hypoxia could inhibit ferroptosis [44, 45], the studies on kidney and lung ischemia–reperfusion injury have shown that hypoxia could promote ferroptosis [46, 47]. Therefore, the regulation of ferroptosis by hypoxia might be different in different tissues and organs.

In order to simulate hypoxia in VECs in PU tissues, VECs were cultured under hypoxia and submitted to gene sequencing. The results of GSEA enrichment analysis suggested that ferroptosis driver genes were significantly enriched in VECs cultured under hypoxia, and vesicle secretion-related genes were significantly enriched in VECs cultured under normoxia. Moreover, abundance and bioinformatics analysis of proteins in EVs suggested that VECs cultured under normoxia could secrete EVs with abundant MFGE8 and oxidoreductase activity. Subsequently, we overexpressed MFGE8 in VECs and observed that the cells could release a large number of EVs containing the bioactive MFGE8 protein, which could be extracted and purified as EVs-MFGE8. EVs-MFGE8 were more effective in inhibiting the ferroptosis of VECs caused by hypoxia than EVs. Studies have shown that MFGE8 was not only rich in EVs and could repair the mitochondrial damage [48] but could also act as an antioxidant that could inhibit the increase in MDA and ROS levels [49]. Moreover, ferroptosis was mainly related to mitochondrial injury caused by the change in oxygen concentration and the increase in ROS levels [50]. So we evaluated whether MFGE8 could affect ferroptosis via regulation of mitochondrial signaling. Our results indicated that overexpressed MFGE8 could effectively inhibit erastin-induced ferroptosis in VECs via inhibiting autophagy. Taking our results (e.g., that MFGE8 reduced ROS levels and inhibited autophagy and ferroptosis) into account, it could be concluded that MFGE8 could inhibit the ferroptosis of VECs via inhibition of autophagy.

EVs have been recognized as a promising drug delivery system in recent years [24, 25]. However, natural EVs are not only small in quantity but also contain few bioactive proteins; thus, obtaining a large amount of EVs for disease treatment may not be possible. As known, CHO cells can effectively synthesize the recombinant protein in large quantities [51], and NPs can also serve as a therapeutic tool carrier for slowing disease progression [52]. Hence, in our study, CHO cells were used to produce a large number of purified recombinant MFGE8 protein, which was isolated and extracted using the magnetic bead method. Finally, we assembled NPs@MFGE8 using silk fibroin NPs and recombinant MFGE8 protein. Current studies have mainly focused on obtaining a large number of EVs as much as possible [53] or studying different types and sources of EVs, such as erythrocyte membrane vesicles [54]. These EVs were not only limited in number but also had complex components, which brought great trouble to the subsequent research on EVs. However, we could effectively solve the above problems by producing the recombinant purified MFGE8 protein and synthesizing EV-derived NPs@MFGE8. EVs have been used as a drug delivery tool for various diseases in experimental and preclinical research, for instance, attenuation of myocardial injury via inhibition of ferroptosis in acute myocardial mice and treatment of inflammatory bone loss [55–57]. Although we knew that EVs could inhibit ferroptosis of VECs through MFGE8 in this study, the role of NPs@MFGE8 in ferroptosis remains unknown. We examined the role of EVs-MFGE8 and NPs@MFGE8 in regulating ferroptosis and found that NPs@MFGE8 and EVs-MFGE8 had similar effects on ferroptosis inhibition. Therefore, NPs@MFGE8 may not only be prepared in large quantities but could also be used in the treatment of ferroptosis-related diseases.

Existing studies showed that the expression level of CD13 was low or that CD13 was not expressed in normal and mature vascular endothelia but was highly abundant on the surface of VECs during tumor neovascularization [29, 58] or in granulation tissues [59]. The pathological characteristics and mechanisms of PU, including granulation tissue formation and hypoxia, were similar to those of tumorigenesis in a tumor microenvironment as well as burned skin. Additionally, MFGE8 was involved in the repair after skin ischemia–reperfusion injury [19] and in accelerating diabetic cutaneous wound healing [23]. Nevertheless, the expression level of CD13 in PU tissues and whether MFGE8 could repair wounds by alleviating the injury to the VECs are still unclear. We found that the CD13 expression was significantly increased in severe PU tissues, whereas the MFGE8 expression was decreased in severe PU tissues as compared with mild PU or normal skin tissues. Therefore, CD13 could be used as a specific target for PU treatment. Moreover, NGR has a high exclusive affinity for CD13 [60]. NGR peptide and silk fibroin NPs contain the amino group (–NH2) [61–63], whereas EDC and NSH can be used as catalysts for the reaction of amino and carboxyl groups [64]. Additionally, HOOC–PEG–COOH can be used as a bridge to cross-link the NGR peptide and silk fibroin NPs so as to construct cross-linked NGR NPs coated with MFGE8 (NGR-NPs@MFGE8), which could specifically target VECs and supplement MFGE8 in PU tissues. To assemble the most efficient sustained-release carrier, we prepared collagen hydrogel, silk fibroin/collagen hydrogel, and silk fibroin hydrogel. Our results indicated that the silk fibroin/collagen hydrogel was the optimal sustained-release carrier based on the analysis of G′/G″, swelling ratio and release efficiency of NPs. The collagen hydrogel had good fluidity that would adhere closely to the wound, but it was easy to lose water, which would lead to slow healing of PU due to too much fluid. Silk fibroin hydrogel has the largest G′ and G″ and high strength and is not closely attached to the wound. Silk fibroin/collagen hydrogel was a combination of the characteristics of collagen hydrogel and silk fibroin hydrogel. It could not only tightly fit the wound but also release NPs at a high rate. Fluorescence results for hypoxia-induced VECs and PU tissues indicated that NGR-NPs@MFGE8 diffused in skin tissues targeted the VECs with high CD13 expression. Therefore, the silk fibroin/collagen hydrogel sustained-release carrier mixed with NGR-NPs@MFGE8 was used in the treatment of PU in rats. The results of our animal experiments proved that NGR-NPs@MFGE8 inhibited autophagy-induced ferroptosis by targeting the VECs in PU tissues in rats, thereby alleviating vascular injury and promoting wound healing. The existing treatments for ulcer repair mainly promote PU regeneration by regulating macrophage polarization, inhibiting ulcer inflammation, enhancing ECM remodeling, or promoting angiogenesis [6, 65]. However, there are few studies on the role of hypoxia in ulcer formation and targeted therapy for PU. In this study, we not only obtained large amounts of EV-derived NGR-NPs@MFGE8 to treat PU but also analyzed the role of hypoxia in PU formation to identify a new mechanism of PU healing, that is, EV-derived NGR-NPs@MFGE8 targeting CD13 of VECs, inhibited ferroptosis of VECs induced by hypoxia and promoted ulcer healing.

## Conclusions

In the current study, we attempted to construct EV-derived NGR-NPs@MFGE8 and investigated its effect on wound healing by targeting VECs in PU tissues. By constructing a silk fibroin/collagen sustained-release carrier that continuously released NGR-NPs@MFGE8 targeting CD13 in VECs, NPs@MFGE8 and EVs-MFGE8 exerted similar effects by inhibiting mitochondrial autophagy-induced ferroptosis and promoting wound healing. This study not only provided an innovative way for treating PU by simulating EVs but also provided a meaningful research direction for the development and application of exosome-derived drugs in the future, such as the use of targeted silk fibroin NPs to coat anti-tumor protein molecules to regulate lactate metabolism or carry tumor- related indicators for early tumor diagnosis, whcih provided a highly promising direction for the study of EV-derived NPs in anti-tumor strategies.

### Supplementary Information

**Additional file 1:** **Figure S1.** Identification of vascular endothelial cells (VECs). **A** Representative immunofluorescence staining of CD31 in VECs. **B** The proportion of CD31 + cells in extracted primary cells was determined by flow cytometry. **Figure S2.** Gene sequencing analysis of VECs treated with normoxia or hypoxia. **A**, **B** The volcano and Venn diagram of VECs treated with normoxia or hypoxia. **C** The differential expression of genes related to the ferroptosis and autophagy of VECs treated with normoxia or hypoxia was analyzed by heat map. **D** KEGG enrichment analysis of upregulated genes in hypoxic VECs compared with normoxic VECs. **E** GO enrichment analysis of upregulated genes in normoxic VECs compared with hypoxic VECs. **Figure S3.** Extracellular vesicles (EVs) inhibit the hypoxia-induced ferroptosis of VECs. **A** Propidium iodide (PI) staining of VECs treated with normoxia, hypoxia, EVs, or hypoxia + EVs was detected by flow cytometry. **B**, **C** The iron, MDA, and GSH levels and mitochondrial changes related to the ferroptosis of VECs treated as above. **D**, **E** Representative western blotting of GPX4 and mean fluorescence intensity (MFI) associated with reactive oxygen species (ROS) levels analyzed in VECs treated with normoxia, hypoxia, EVs, or hypoxia + EVs. ns: *p* > 0.05; *p < 0.05; **p < 0.01; ***p < 0.001. **Figure S4.** Ferroptosis was enhanced by autophagy in VECs. **A** KEGG enrichment analysis of all differential genes in VECs treated with or without hypoxia. **B**, **C** Representative western blot analysis of LC3A/B, ACSL4, GPX4, P53, P62, and lipid peroxidation in VECs treated with dimethyl sulfoxide (DMSO), erastin, or erastin + 3-methyladenine. **D**, **E** Iron, MDA, and GSH levels and mitochondrial changes associated with the ferroptosis of VECs treated as above. **Figure S5.** MFGE8 inhibited ferroptosis by diminishing autophagy in VECs. **A** Correlation analysis of MFGE8, ferroptosis-related proteins, and autophagy-related proteins. **B**–**D** Representative western blot analysis of P53, ACSL4, GPX4, LC3A/B, and MFGE8, reactive oxygen species (ROS) detected by flow cytometry, and invasive capability detected by transwell assay of VECs treated with dimethyl sulfoxide (DMSO), erastin, Lenti-MFGE8, or erastin + Lenti-MFGE8. **J**, **K** Representative immunofluorescence staining of MFGE8/LC3B and GPX4/ACSL4 in VECs treated as above. NC: normal control. ns: *p* > 0.05; *p < 0.05; **p < 0.01; ***p < 0.001. **Figure S6.** Characteristics of MFGE8 released from NPs@MFGE8. The rate of MFGE8 released from NPs@MFGE8 was examined by HPLC of the NPs@MFGE8 on days 1, 2, 3, 4, 5, 6, and 7. **Figure S7.** Detection and analysis of the NPs clearance uptake by VECs. **A** The preservation of RBITC-NPs in VECs was observed by fluorescence microscopy at days 0, 1, 3, 5, and 7. **B** The mean fluorescence intensity (MFI) of VECs after uptake of RBITC-NPs was statistically analyzed at days 0 1, 3, 5, and 7. **C** The clearance rate of NPs and NPs@MFGE8 in VECs was statistically analyzed at days 1, 3, 5, and 7. ns: *p* > 0.05. **Figure S8.** The critical concentration of NPs@MFGE8 in enhancing VEC viability. The CCK-8 assay was used to further illustrate the critical concentration of NPs@MFGE8. Approximately 1 ml of purified MFGE8 protein (1 mg/ml) was added to 1 ml of silk fibroin solution (10 mg/ml) with a mass ratio of 1:10 to prepare the NPs@MFGE8. The VECs were cultured under hypoxic conditions and subsequently treated with different concentrations of NPs@MFGE8 (100, 250, 500, 750, or 1000 μg/ml). The VECs viability was detected by the CCK-8 assay, and the critical concentration of NPs@MFGE8 in enhancing VEC viability might be 500 μg/ml in vitro. ns: *p* > 0.05; *p < 0.05. **Figure S9.** The concentration of MFGE8 in NPs@MFGE8 (500 μg/ml) was determined. **A** The detection of MFGE8 standard solution (0.2 mg/ml) by HPLC. **B** The detection of MFGE8 in NPs@MFGE8 by HPLC after lysis with LiBr. The peaks of the MFGE8 protein were 2.053 min and 2.127 min, respectively. **C** The concentration of MFGE8 in NPs@MFGE8 (500 μg/ml) was calculated according to the concentration and the area of the waveform of MFGE8 (0.2 mg/ml). **Figure S10.** The degradation rate of the Silk fibroin/collagen hydrogel. After treated with collagenase I and II, trypsin, and neutral proteinase for 10 days, the hydrogel carriers were gradually degraded. **Figure S11.** The effect of hydrogel sustained-release carrier on promoting PU healing. **A**, **B** Wound size and its statistical analysis at days 0, 1, 3, 6, 9, and 12. ns (green): PU + Gels-NPs *vs.* PU + Gels; ns (yellow) and *(yellow): PU + Gels-NPs *vs.* PU + Gels-NPs@MFGE8. **C** Masson staining was conducted to detect wound healing in the PU + Gels, PU + Gels-NPs, and PU + Gels-NPs@MFGE8 groups. **Figure S12.** Immunohistochemistry of parkin, pink1, MFGE8, and GPX4 in VECs in rats' skin tissues, which were divided into NC group, PU group, PU + silk fibroin/collagen hydrogel (Gels) group, PU + silk fibroin/collagen hydrogel-NPs@MFGE8 (Gels-NPs@MFGE8) group, and PU + silk fibroin/collagen hydrogel-NGR-NPs@MFGE8 (Gels-NGR-NPs@MFGE8) group. **Figure S13.** Analysis of the expression levels of macrophage marker protein CD14 and fibroblast marker protein α-SMA in skin tissues. **A**, **B** Immunohistochemical staining of macrophage marker protein CD14 and statistical analysis of macrophage numbers in the NC group, PU group, PU + Gels group, PU + Gels-NPs@MFGE8 group, and PU + Gels-NGR-NPs@MFGE8 group. **C**, **D** Immunohistochemical staining of fibroblast marker protein α-SMA and statistical analysis of fibroblast numbers in the five groups above. ns: *p* > 0.05; *p < 0.05; **p < 0.01; ***p < 0.001.

## Data Availability

The data used to support the findings of this study are available from the corresponding author on request.

## Abbreviations

MFGE8: Milk fat globule EGF factor 8
PU: Pressure ulcers
VECs: Vascular endothelial cells
NPs: Nanoparticles
EVs: Extracellular vesicles
GAPDH: Glyceraldehyde 3-phosphate dehydrogenase
TEM: Transmission electron microscopy
SEM: Scanning Electronic Microscopy
NTA: Nanoparticle tracking analysis
GO: Gene ontology
KEGG: Kyoto Encyclopedia of Genes and Genomes
GSEA: Gene set enrichment analysis
HPLC: High-performance liquid chromatography
Gels: Silk fibroin/collagen hydrogels
CCK-8: Cell Counting Kit-8
Hypo: Hypoxia
