# Supplementary figures and images for: Extracellular vesicle-derived silk fibroin nanoparticles loaded with MFGE8 accelerate skin ulcer healing by targeting the vascular endothelial cells
Source: J Nanobiotechnology. 2023 Nov 29;21:455. doi: 10.1186/s12951-023-02185-7 (PMC10685683; doi:10.1186/s12951-023-02185-7)

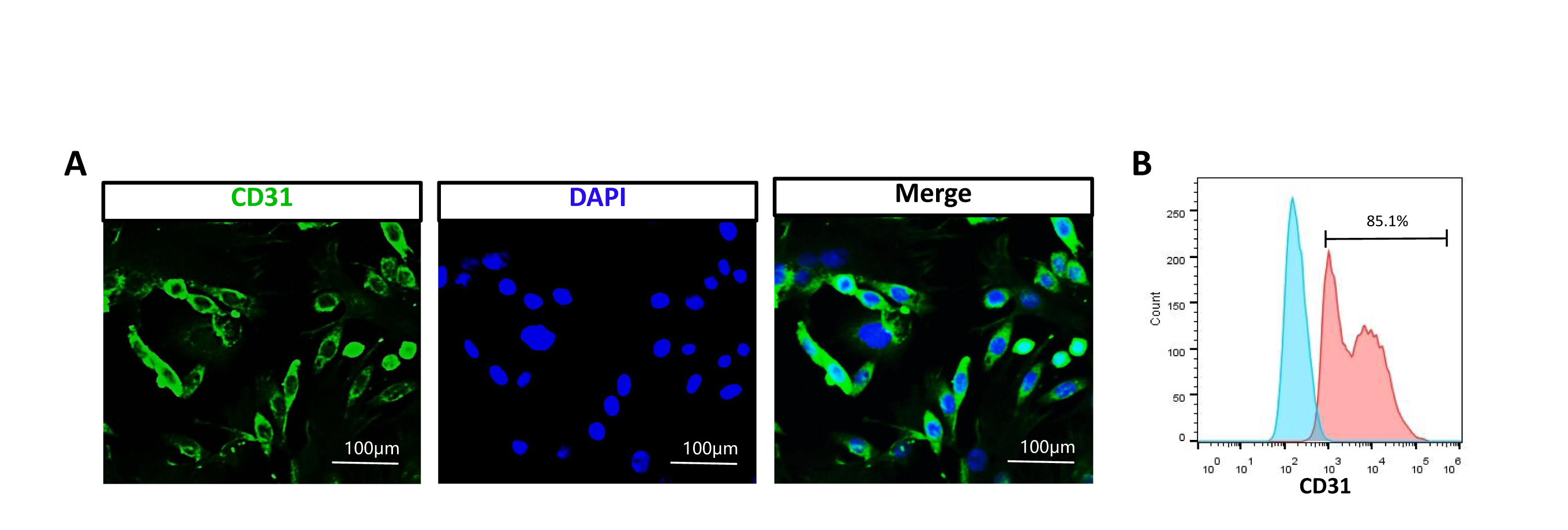

Supplement: Supplementary file 1 — Additional file 1: Figure S1. Identification of vascular endothelial cells (VECs). A Representative immunofluorescence staining of CD31 in VECs. B The proportion of CD31 + cells in extracted primary cells was determined by flow cytometry. Figure S2. Gene sequencing analysis of VECs treated with normoxia or hypoxia. A, B The volcano and Venn diagram of VECs treated with normoxia or hypoxia. C The differential expression of genes related to the ferroptosis and autophagy of VECs treated with normoxia or hypoxia was analyzed by heat map. D KEGG enrichment analysis of upregulated genes in hypoxic VECs compared with normoxic VECs. E GO enrichment analysis of upregulated genes in normoxic VECs compared with hypoxic VECs. Figure S3. Extracellular vesicles (EVs) inhibit the hypoxia-induced ferroptosis of VECs. A Propidium iodide (PI) staining of VECs treated with normoxia, hypoxia, EVs, or hypoxia + EVs was detected by flow cytometry. B, C The iron, MDA, and GSH levels and mitochondrial changes related to the ferroptosis of VECs treated as above. D, E Representative western blotting of GPX4 and mean fluorescence intensity (MFI) associated with reactive oxygen species (ROS) levels analyzed in VECs treated with normoxia, hypoxia, EVs, or hypoxia + EVs. ns: p > 0.05; *p < 0.05; **p < 0.01; ***p < 0.001. Figure S4. Ferroptosis was enhanced by autophagy in VECs. A KEGG enrichment analysis of all differential genes in VECs treated with or without hypoxia. B, C Representative western blot analysis of LC3A/B, ACSL4, GPX4, P53, P62, and lipid peroxidation in VECs treated with dimethyl sulfoxide (DMSO), erastin, or erastin + 3-methyladenine. D, E Iron, MDA, and GSH levels and mitochondrial changes associated with the ferroptosis of VECs treated as above. Figure S5. MFGE8 inhibited ferroptosis by diminishing autophagy in VECs. A Correlation analysis of MFGE8, ferroptosis-related proteins, and autophagy-related proteins. B–D Representative western blot analysis of P53, ACSL4, GPX4, LC3A [file 12951_2023_2185_MOESM1_ESM.zip › figure S1.jpg]

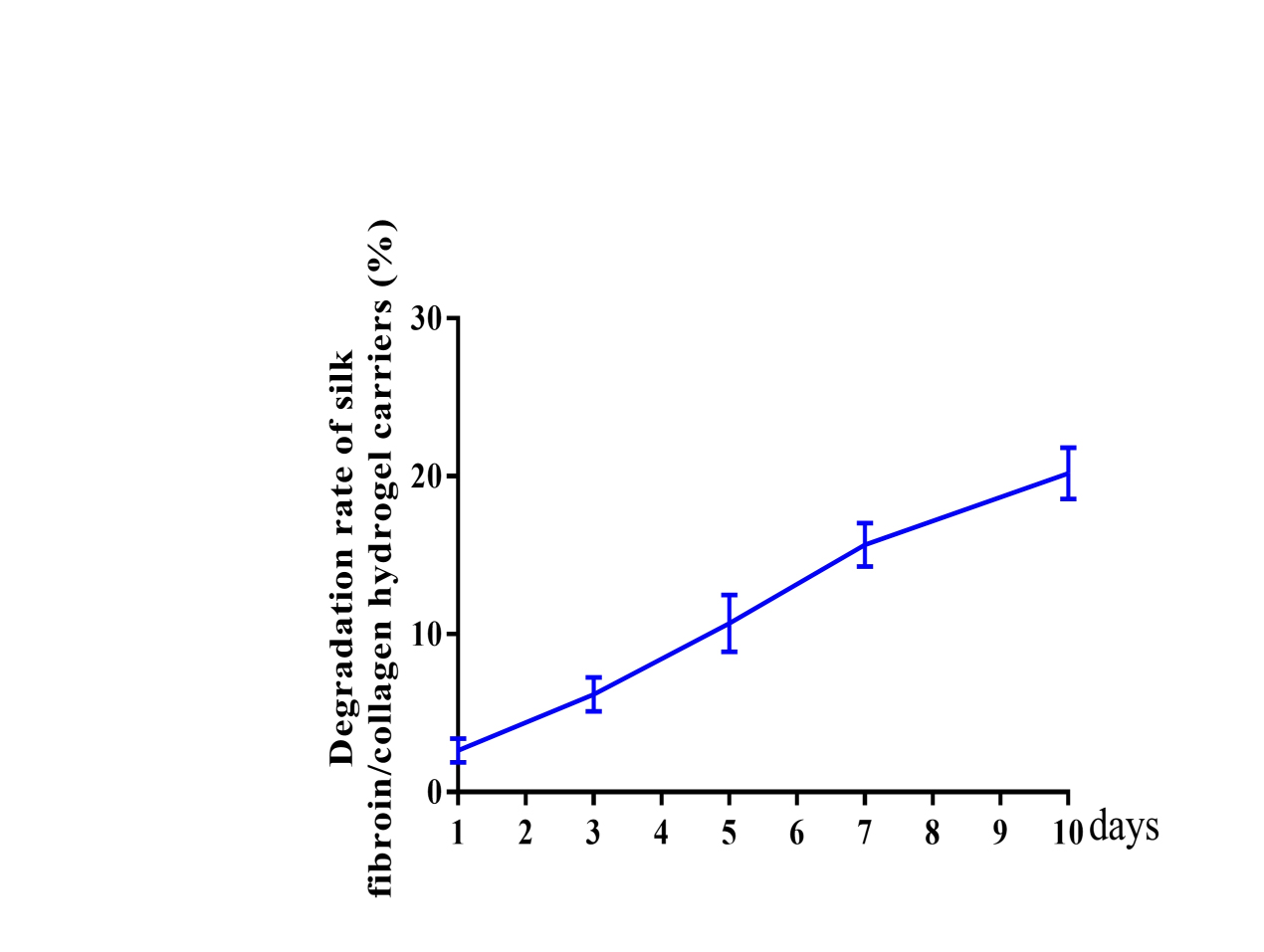

Supplement: Supplementary file 1 — Additional file 1: Figure S1. Identification of vascular endothelial cells (VECs). A Representative immunofluorescence staining of CD31 in VECs. B The proportion of CD31 + cells in extracted primary cells was determined by flow cytometry. Figure S2. Gene sequencing analysis of VECs treated with normoxia or hypoxia. A, B The volcano and Venn diagram of VECs treated with normoxia or hypoxia. C The differential expression of genes related to the ferroptosis and autophagy of VECs treated with normoxia or hypoxia was analyzed by heat map. D KEGG enrichment analysis of upregulated genes in hypoxic VECs compared with normoxic VECs. E GO enrichment analysis of upregulated genes in normoxic VECs compared with hypoxic VECs. Figure S3. Extracellular vesicles (EVs) inhibit the hypoxia-induced ferroptosis of VECs. A Propidium iodide (PI) staining of VECs treated with normoxia, hypoxia, EVs, or hypoxia + EVs was detected by flow cytometry. B, C The iron, MDA, and GSH levels and mitochondrial changes related to the ferroptosis of VECs treated as above. D, E Representative western blotting of GPX4 and mean fluorescence intensity (MFI) associated with reactive oxygen species (ROS) levels analyzed in VECs treated with normoxia, hypoxia, EVs, or hypoxia + EVs. ns: p > 0.05; *p < 0.05; **p < 0.01; ***p < 0.001. Figure S4. Ferroptosis was enhanced by autophagy in VECs. A KEGG enrichment analysis of all differential genes in VECs treated with or without hypoxia. B, C Representative western blot analysis of LC3A/B, ACSL4, GPX4, P53, P62, and lipid peroxidation in VECs treated with dimethyl sulfoxide (DMSO), erastin, or erastin + 3-methyladenine. D, E Iron, MDA, and GSH levels and mitochondrial changes associated with the ferroptosis of VECs treated as above. Figure S5. MFGE8 inhibited ferroptosis by diminishing autophagy in VECs. A Correlation analysis of MFGE8, ferroptosis-related proteins, and autophagy-related proteins. B–D Representative western blot analysis of P53, ACSL4, GPX4, LC3A [file 12951_2023_2185_MOESM1_ESM.zip › figure S10.jpg]

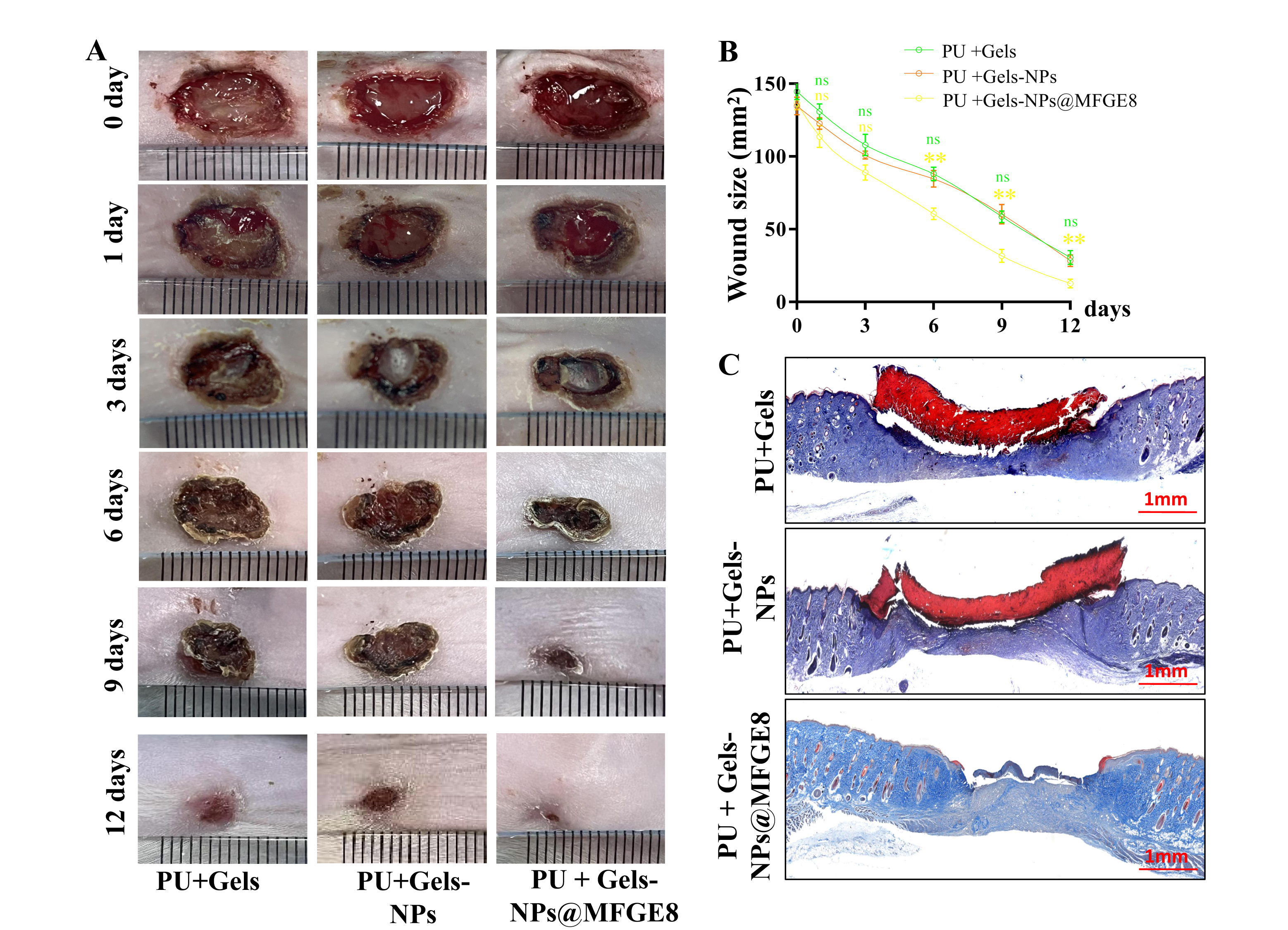

Supplement: Supplementary file 1 — Additional file 1: Figure S1. Identification of vascular endothelial cells (VECs). A Representative immunofluorescence staining of CD31 in VECs. B The proportion of CD31 + cells in extracted primary cells was determined by flow cytometry. Figure S2. Gene sequencing analysis of VECs treated with normoxia or hypoxia. A, B The volcano and Venn diagram of VECs treated with normoxia or hypoxia. C The differential expression of genes related to the ferroptosis and autophagy of VECs treated with normoxia or hypoxia was analyzed by heat map. D KEGG enrichment analysis of upregulated genes in hypoxic VECs compared with normoxic VECs. E GO enrichment analysis of upregulated genes in normoxic VECs compared with hypoxic VECs. Figure S3. Extracellular vesicles (EVs) inhibit the hypoxia-induced ferroptosis of VECs. A Propidium iodide (PI) staining of VECs treated with normoxia, hypoxia, EVs, or hypoxia + EVs was detected by flow cytometry. B, C The iron, MDA, and GSH levels and mitochondrial changes related to the ferroptosis of VECs treated as above. D, E Representative western blotting of GPX4 and mean fluorescence intensity (MFI) associated with reactive oxygen species (ROS) levels analyzed in VECs treated with normoxia, hypoxia, EVs, or hypoxia + EVs. ns: p > 0.05; *p < 0.05; **p < 0.01; ***p < 0.001. Figure S4. Ferroptosis was enhanced by autophagy in VECs. A KEGG enrichment analysis of all differential genes in VECs treated with or without hypoxia. B, C Representative western blot analysis of LC3A/B, ACSL4, GPX4, P53, P62, and lipid peroxidation in VECs treated with dimethyl sulfoxide (DMSO), erastin, or erastin + 3-methyladenine. D, E Iron, MDA, and GSH levels and mitochondrial changes associated with the ferroptosis of VECs treated as above. Figure S5. MFGE8 inhibited ferroptosis by diminishing autophagy in VECs. A Correlation analysis of MFGE8, ferroptosis-related proteins, and autophagy-related proteins. B–D Representative western blot analysis of P53, ACSL4, GPX4, LC3A [file 12951_2023_2185_MOESM1_ESM.zip › figure S11.jpg]

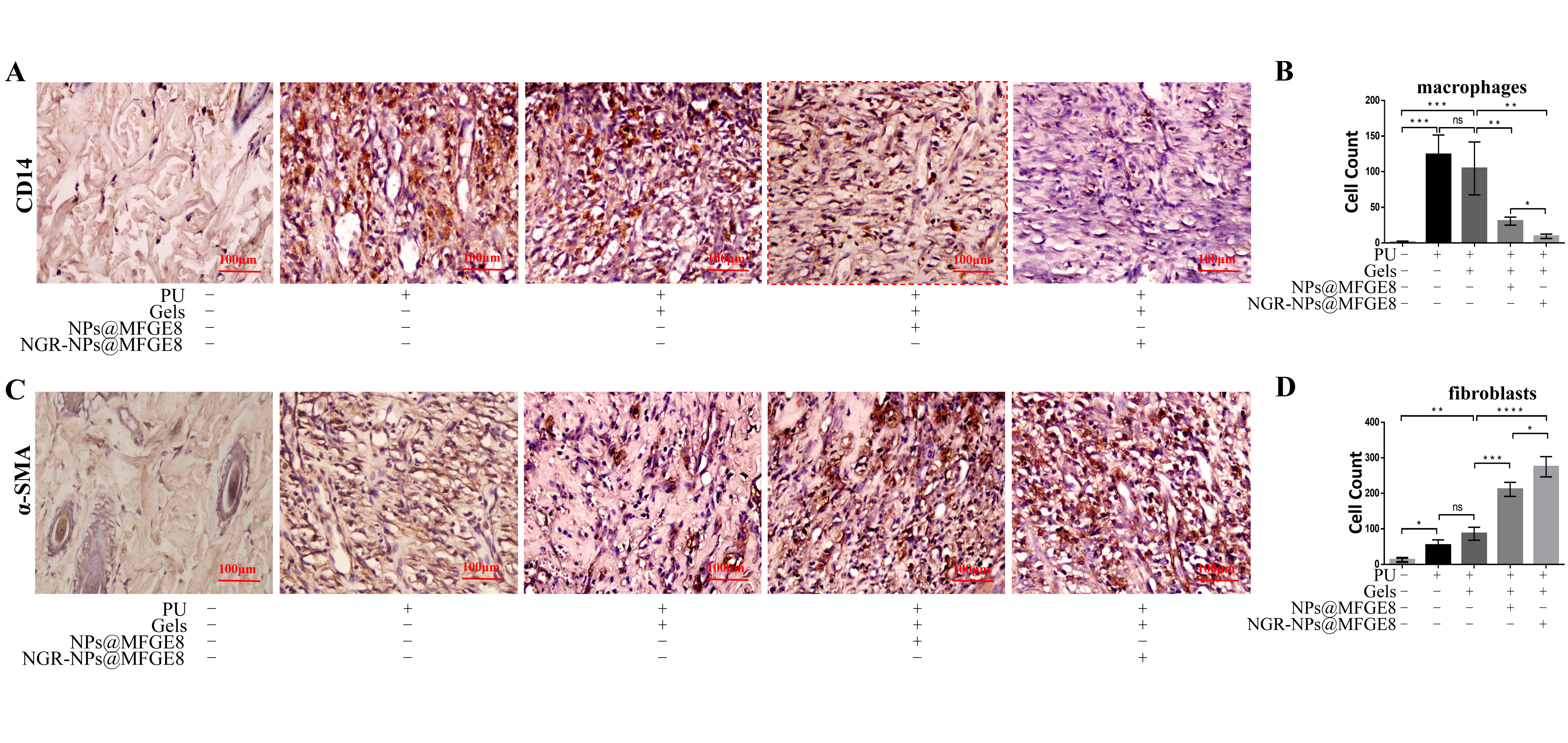

Supplement: Supplementary file 1 — Additional file 1: Figure S1. Identification of vascular endothelial cells (VECs). A Representative immunofluorescence staining of CD31 in VECs. B The proportion of CD31 + cells in extracted primary cells was determined by flow cytometry. Figure S2. Gene sequencing analysis of VECs treated with normoxia or hypoxia. A, B The volcano and Venn diagram of VECs treated with normoxia or hypoxia. C The differential expression of genes related to the ferroptosis and autophagy of VECs treated with normoxia or hypoxia was analyzed by heat map. D KEGG enrichment analysis of upregulated genes in hypoxic VECs compared with normoxic VECs. E GO enrichment analysis of upregulated genes in normoxic VECs compared with hypoxic VECs. Figure S3. Extracellular vesicles (EVs) inhibit the hypoxia-induced ferroptosis of VECs. A Propidium iodide (PI) staining of VECs treated with normoxia, hypoxia, EVs, or hypoxia + EVs was detected by flow cytometry. B, C The iron, MDA, and GSH levels and mitochondrial changes related to the ferroptosis of VECs treated as above. D, E Representative western blotting of GPX4 and mean fluorescence intensity (MFI) associated with reactive oxygen species (ROS) levels analyzed in VECs treated with normoxia, hypoxia, EVs, or hypoxia + EVs. ns: p > 0.05; *p < 0.05; **p < 0.01; ***p < 0.001. Figure S4. Ferroptosis was enhanced by autophagy in VECs. A KEGG enrichment analysis of all differential genes in VECs treated with or without hypoxia. B, C Representative western blot analysis of LC3A/B, ACSL4, GPX4, P53, P62, and lipid peroxidation in VECs treated with dimethyl sulfoxide (DMSO), erastin, or erastin + 3-methyladenine. D, E Iron, MDA, and GSH levels and mitochondrial changes associated with the ferroptosis of VECs treated as above. Figure S5. MFGE8 inhibited ferroptosis by diminishing autophagy in VECs. A Correlation analysis of MFGE8, ferroptosis-related proteins, and autophagy-related proteins. B–D Representative western blot analysis of P53, ACSL4, GPX4, LC3A [file 12951_2023_2185_MOESM1_ESM.zip › figure S13.jpg]

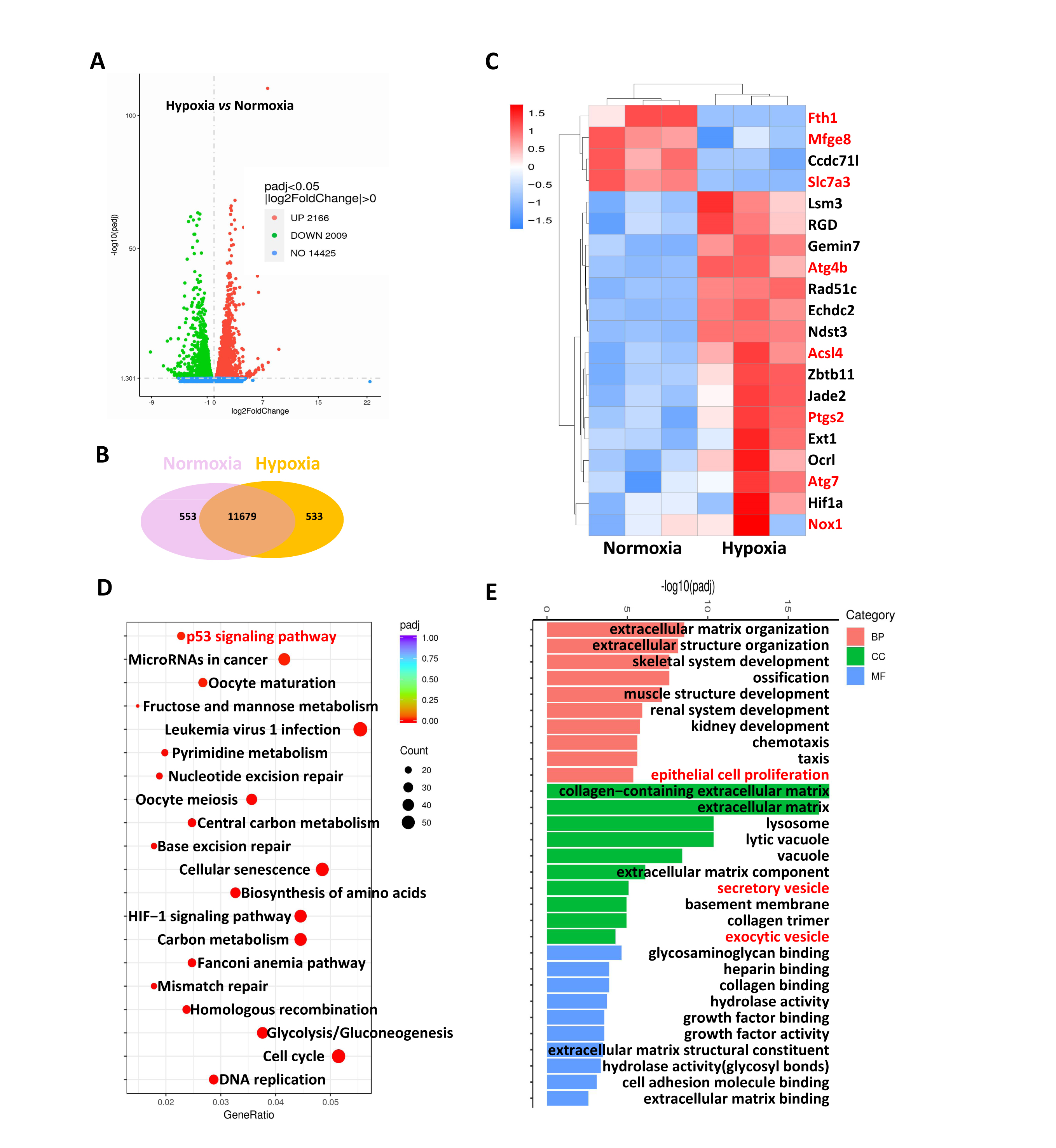

Supplement: Supplementary file 1 — Additional file 1: Figure S1. Identification of vascular endothelial cells (VECs). A Representative immunofluorescence staining of CD31 in VECs. B The proportion of CD31 + cells in extracted primary cells was determined by flow cytometry. Figure S2. Gene sequencing analysis of VECs treated with normoxia or hypoxia. A, B The volcano and Venn diagram of VECs treated with normoxia or hypoxia. C The differential expression of genes related to the ferroptosis and autophagy of VECs treated with normoxia or hypoxia was analyzed by heat map. D KEGG enrichment analysis of upregulated genes in hypoxic VECs compared with normoxic VECs. E GO enrichment analysis of upregulated genes in normoxic VECs compared with hypoxic VECs. Figure S3. Extracellular vesicles (EVs) inhibit the hypoxia-induced ferroptosis of VECs. A Propidium iodide (PI) staining of VECs treated with normoxia, hypoxia, EVs, or hypoxia + EVs was detected by flow cytometry. B, C The iron, MDA, and GSH levels and mitochondrial changes related to the ferroptosis of VECs treated as above. D, E Representative western blotting of GPX4 and mean fluorescence intensity (MFI) associated with reactive oxygen species (ROS) levels analyzed in VECs treated with normoxia, hypoxia, EVs, or hypoxia + EVs. ns: p > 0.05; *p < 0.05; **p < 0.01; ***p < 0.001. Figure S4. Ferroptosis was enhanced by autophagy in VECs. A KEGG enrichment analysis of all differential genes in VECs treated with or without hypoxia. B, C Representative western blot analysis of LC3A/B, ACSL4, GPX4, P53, P62, and lipid peroxidation in VECs treated with dimethyl sulfoxide (DMSO), erastin, or erastin + 3-methyladenine. D, E Iron, MDA, and GSH levels and mitochondrial changes associated with the ferroptosis of VECs treated as above. Figure S5. MFGE8 inhibited ferroptosis by diminishing autophagy in VECs. A Correlation analysis of MFGE8, ferroptosis-related proteins, and autophagy-related proteins. B–D Representative western blot analysis of P53, ACSL4, GPX4, LC3A [file 12951_2023_2185_MOESM1_ESM.zip › figure S2.jpg]

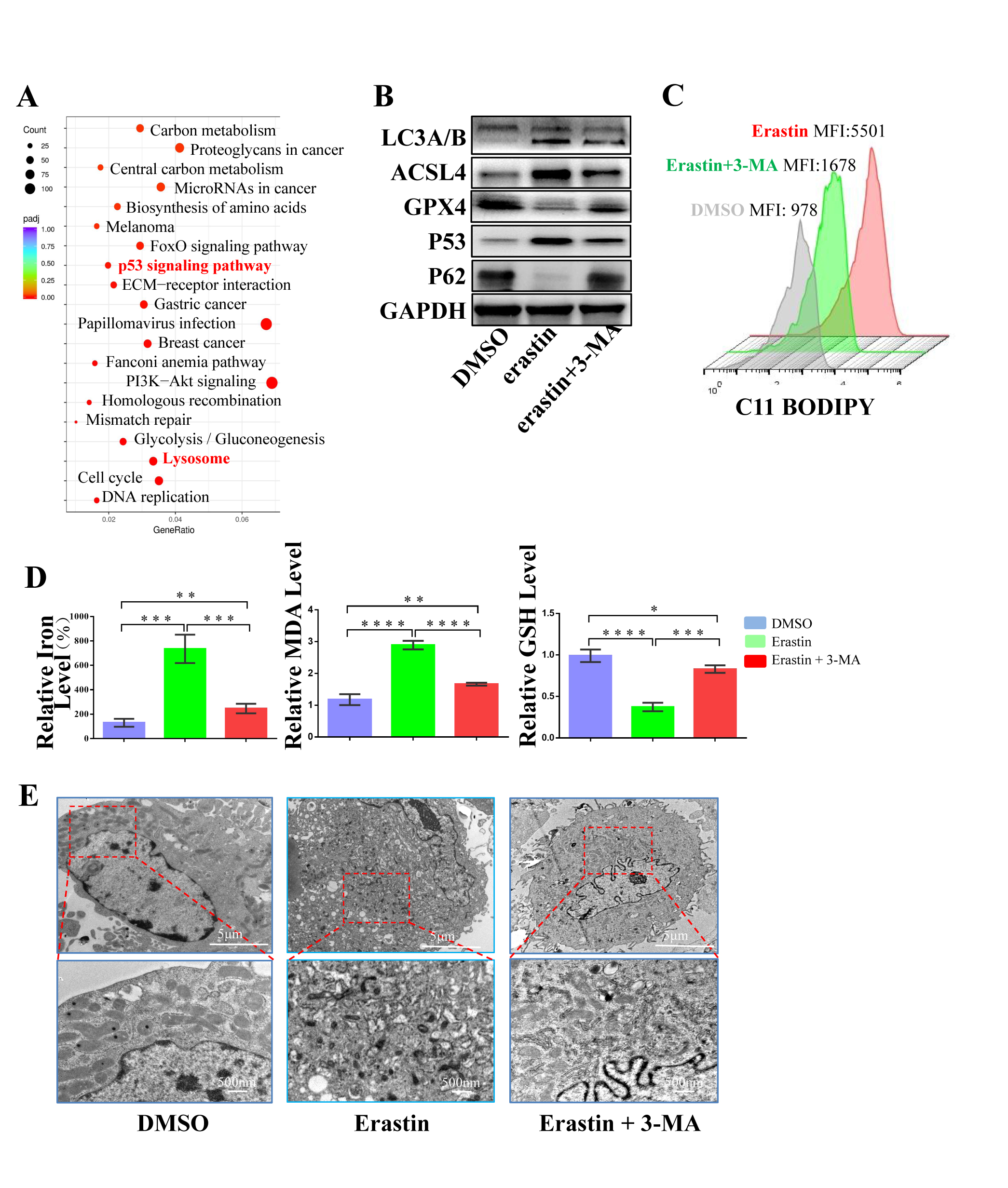

Supplement: Supplementary file 1 — Additional file 1: Figure S1. Identification of vascular endothelial cells (VECs). A Representative immunofluorescence staining of CD31 in VECs. B The proportion of CD31 + cells in extracted primary cells was determined by flow cytometry. Figure S2. Gene sequencing analysis of VECs treated with normoxia or hypoxia. A, B The volcano and Venn diagram of VECs treated with normoxia or hypoxia. C The differential expression of genes related to the ferroptosis and autophagy of VECs treated with normoxia or hypoxia was analyzed by heat map. D KEGG enrichment analysis of upregulated genes in hypoxic VECs compared with normoxic VECs. E GO enrichment analysis of upregulated genes in normoxic VECs compared with hypoxic VECs. Figure S3. Extracellular vesicles (EVs) inhibit the hypoxia-induced ferroptosis of VECs. A Propidium iodide (PI) staining of VECs treated with normoxia, hypoxia, EVs, or hypoxia + EVs was detected by flow cytometry. B, C The iron, MDA, and GSH levels and mitochondrial changes related to the ferroptosis of VECs treated as above. D, E Representative western blotting of GPX4 and mean fluorescence intensity (MFI) associated with reactive oxygen species (ROS) levels analyzed in VECs treated with normoxia, hypoxia, EVs, or hypoxia + EVs. ns: p > 0.05; *p < 0.05; **p < 0.01; ***p < 0.001. Figure S4. Ferroptosis was enhanced by autophagy in VECs. A KEGG enrichment analysis of all differential genes in VECs treated with or without hypoxia. B, C Representative western blot analysis of LC3A/B, ACSL4, GPX4, P53, P62, and lipid peroxidation in VECs treated with dimethyl sulfoxide (DMSO), erastin, or erastin + 3-methyladenine. D, E Iron, MDA, and GSH levels and mitochondrial changes associated with the ferroptosis of VECs treated as above. Figure S5. MFGE8 inhibited ferroptosis by diminishing autophagy in VECs. A Correlation analysis of MFGE8, ferroptosis-related proteins, and autophagy-related proteins. B–D Representative western blot analysis of P53, ACSL4, GPX4, LC3A [file 12951_2023_2185_MOESM1_ESM.zip › figure S4.jpg]

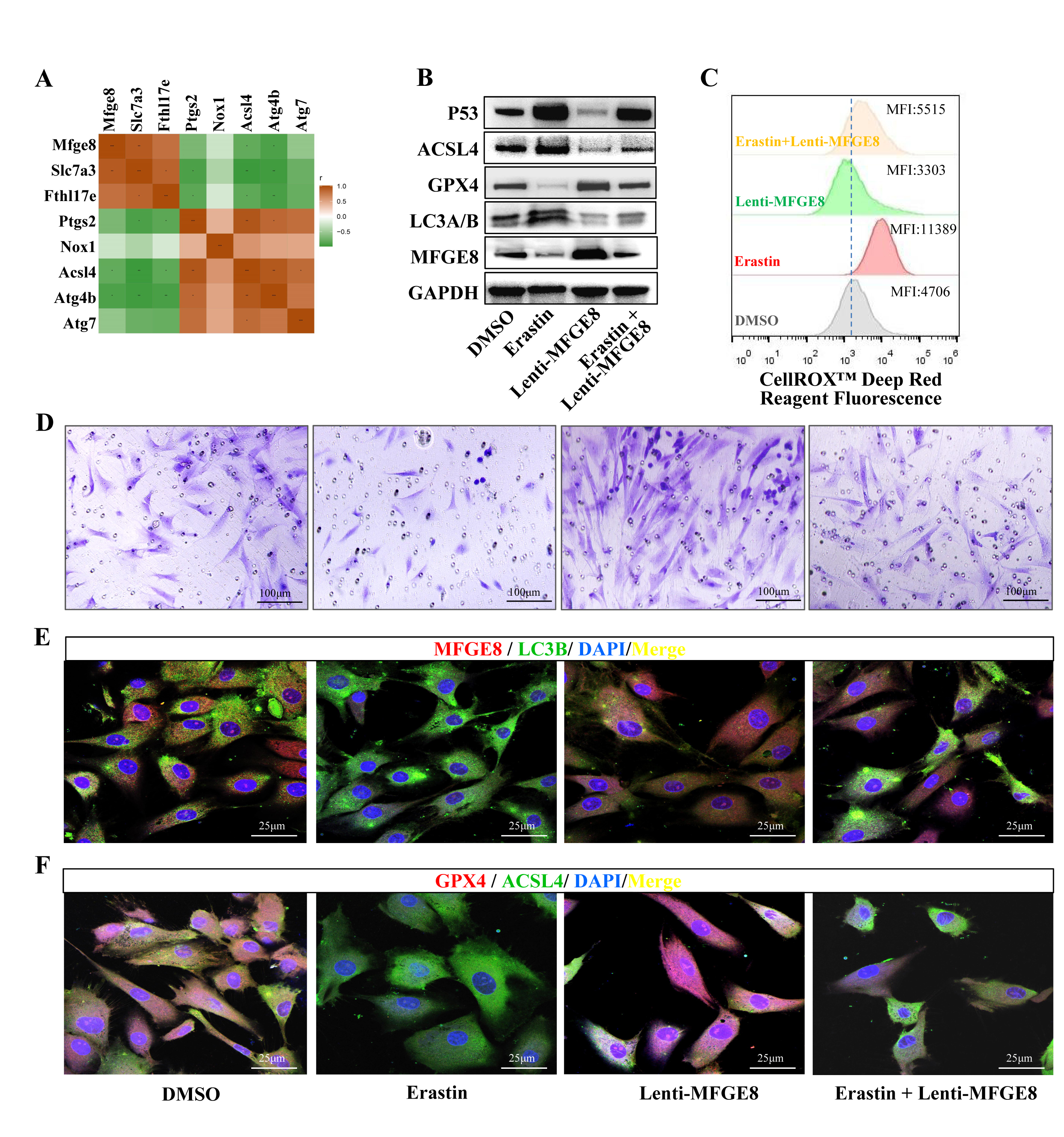

Supplement: Supplementary file 1 — Additional file 1: Figure S1. Identification of vascular endothelial cells (VECs). A Representative immunofluorescence staining of CD31 in VECs. B The proportion of CD31 + cells in extracted primary cells was determined by flow cytometry. Figure S2. Gene sequencing analysis of VECs treated with normoxia or hypoxia. A, B The volcano and Venn diagram of VECs treated with normoxia or hypoxia. C The differential expression of genes related to the ferroptosis and autophagy of VECs treated with normoxia or hypoxia was analyzed by heat map. D KEGG enrichment analysis of upregulated genes in hypoxic VECs compared with normoxic VECs. E GO enrichment analysis of upregulated genes in normoxic VECs compared with hypoxic VECs. Figure S3. Extracellular vesicles (EVs) inhibit the hypoxia-induced ferroptosis of VECs. A Propidium iodide (PI) staining of VECs treated with normoxia, hypoxia, EVs, or hypoxia + EVs was detected by flow cytometry. B, C The iron, MDA, and GSH levels and mitochondrial changes related to the ferroptosis of VECs treated as above. D, E Representative western blotting of GPX4 and mean fluorescence intensity (MFI) associated with reactive oxygen species (ROS) levels analyzed in VECs treated with normoxia, hypoxia, EVs, or hypoxia + EVs. ns: p > 0.05; *p < 0.05; **p < 0.01; ***p < 0.001. Figure S4. Ferroptosis was enhanced by autophagy in VECs. A KEGG enrichment analysis of all differential genes in VECs treated with or without hypoxia. B, C Representative western blot analysis of LC3A/B, ACSL4, GPX4, P53, P62, and lipid peroxidation in VECs treated with dimethyl sulfoxide (DMSO), erastin, or erastin + 3-methyladenine. D, E Iron, MDA, and GSH levels and mitochondrial changes associated with the ferroptosis of VECs treated as above. Figure S5. MFGE8 inhibited ferroptosis by diminishing autophagy in VECs. A Correlation analysis of MFGE8, ferroptosis-related proteins, and autophagy-related proteins. B–D Representative western blot analysis of P53, ACSL4, GPX4, LC3A [file 12951_2023_2185_MOESM1_ESM.zip › figure S5.jpg]

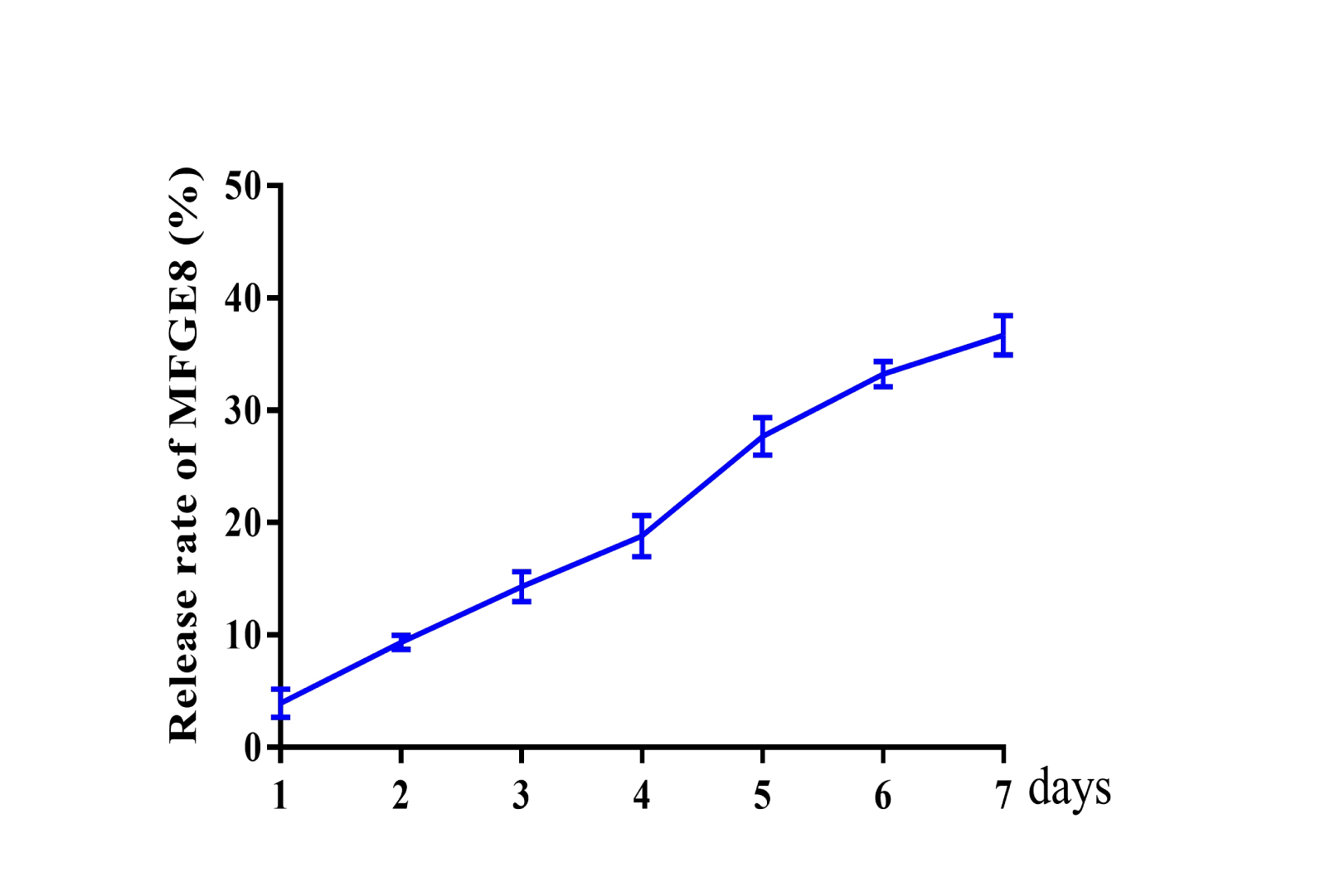

Supplement: Supplementary file 1 — Additional file 1: Figure S1. Identification of vascular endothelial cells (VECs). A Representative immunofluorescence staining of CD31 in VECs. B The proportion of CD31 + cells in extracted primary cells was determined by flow cytometry. Figure S2. Gene sequencing analysis of VECs treated with normoxia or hypoxia. A, B The volcano and Venn diagram of VECs treated with normoxia or hypoxia. C The differential expression of genes related to the ferroptosis and autophagy of VECs treated with normoxia or hypoxia was analyzed by heat map. D KEGG enrichment analysis of upregulated genes in hypoxic VECs compared with normoxic VECs. E GO enrichment analysis of upregulated genes in normoxic VECs compared with hypoxic VECs. Figure S3. Extracellular vesicles (EVs) inhibit the hypoxia-induced ferroptosis of VECs. A Propidium iodide (PI) staining of VECs treated with normoxia, hypoxia, EVs, or hypoxia + EVs was detected by flow cytometry. B, C The iron, MDA, and GSH levels and mitochondrial changes related to the ferroptosis of VECs treated as above. D, E Representative western blotting of GPX4 and mean fluorescence intensity (MFI) associated with reactive oxygen species (ROS) levels analyzed in VECs treated with normoxia, hypoxia, EVs, or hypoxia + EVs. ns: p > 0.05; *p < 0.05; **p < 0.01; ***p < 0.001. Figure S4. Ferroptosis was enhanced by autophagy in VECs. A KEGG enrichment analysis of all differential genes in VECs treated with or without hypoxia. B, C Representative western blot analysis of LC3A/B, ACSL4, GPX4, P53, P62, and lipid peroxidation in VECs treated with dimethyl sulfoxide (DMSO), erastin, or erastin + 3-methyladenine. D, E Iron, MDA, and GSH levels and mitochondrial changes associated with the ferroptosis of VECs treated as above. Figure S5. MFGE8 inhibited ferroptosis by diminishing autophagy in VECs. A Correlation analysis of MFGE8, ferroptosis-related proteins, and autophagy-related proteins. B–D Representative western blot analysis of P53, ACSL4, GPX4, LC3A [file 12951_2023_2185_MOESM1_ESM.zip › figure S6.jpg]

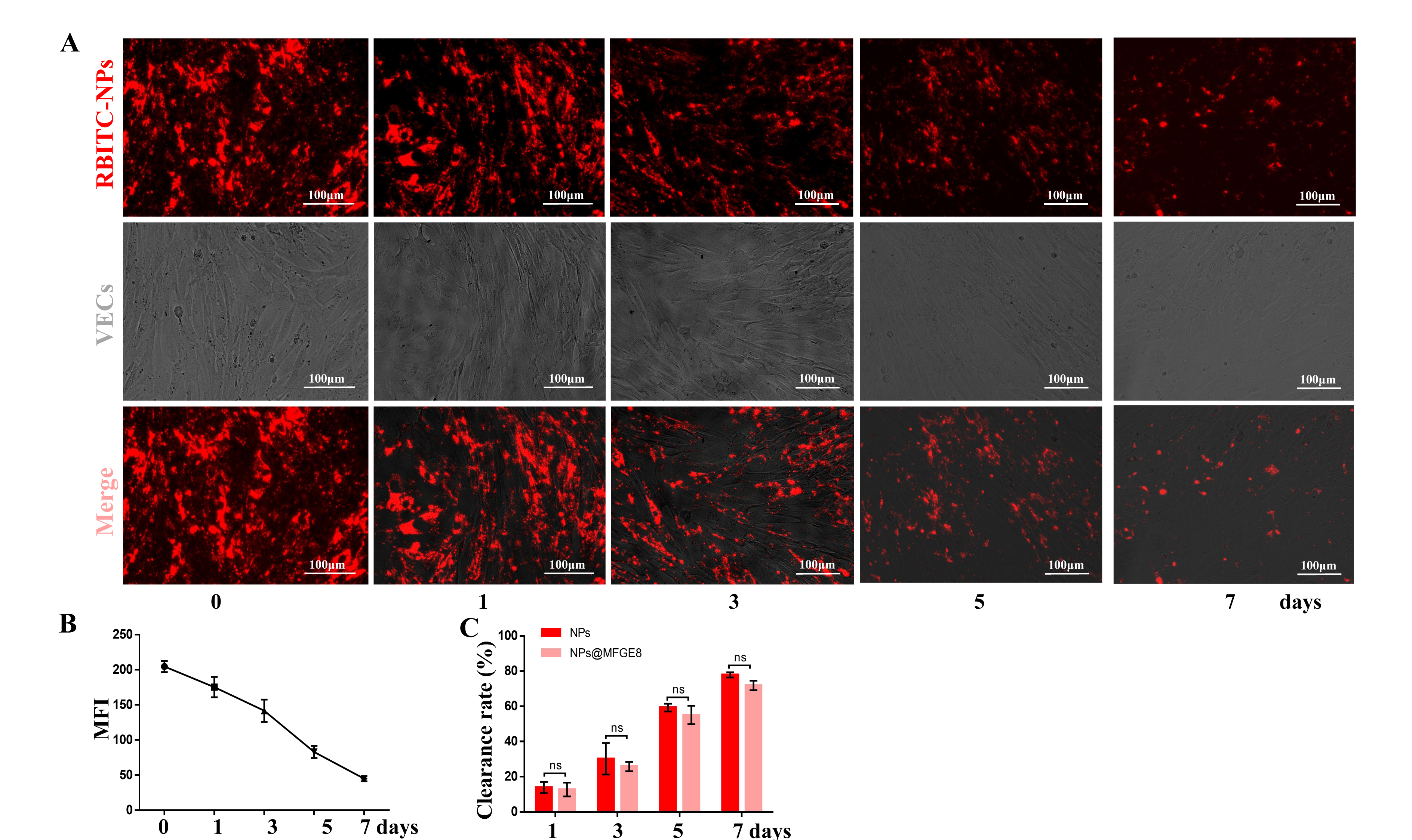

Supplement: Supplementary file 1 — Additional file 1: Figure S1. Identification of vascular endothelial cells (VECs). A Representative immunofluorescence staining of CD31 in VECs. B The proportion of CD31 + cells in extracted primary cells was determined by flow cytometry. Figure S2. Gene sequencing analysis of VECs treated with normoxia or hypoxia. A, B The volcano and Venn diagram of VECs treated with normoxia or hypoxia. C The differential expression of genes related to the ferroptosis and autophagy of VECs treated with normoxia or hypoxia was analyzed by heat map. D KEGG enrichment analysis of upregulated genes in hypoxic VECs compared with normoxic VECs. E GO enrichment analysis of upregulated genes in normoxic VECs compared with hypoxic VECs. Figure S3. Extracellular vesicles (EVs) inhibit the hypoxia-induced ferroptosis of VECs. A Propidium iodide (PI) staining of VECs treated with normoxia, hypoxia, EVs, or hypoxia + EVs was detected by flow cytometry. B, C The iron, MDA, and GSH levels and mitochondrial changes related to the ferroptosis of VECs treated as above. D, E Representative western blotting of GPX4 and mean fluorescence intensity (MFI) associated with reactive oxygen species (ROS) levels analyzed in VECs treated with normoxia, hypoxia, EVs, or hypoxia + EVs. ns: p > 0.05; *p < 0.05; **p < 0.01; ***p < 0.001. Figure S4. Ferroptosis was enhanced by autophagy in VECs. A KEGG enrichment analysis of all differential genes in VECs treated with or without hypoxia. B, C Representative western blot analysis of LC3A/B, ACSL4, GPX4, P53, P62, and lipid peroxidation in VECs treated with dimethyl sulfoxide (DMSO), erastin, or erastin + 3-methyladenine. D, E Iron, MDA, and GSH levels and mitochondrial changes associated with the ferroptosis of VECs treated as above. Figure S5. MFGE8 inhibited ferroptosis by diminishing autophagy in VECs. A Correlation analysis of MFGE8, ferroptosis-related proteins, and autophagy-related proteins. B–D Representative western blot analysis of P53, ACSL4, GPX4, LC3A [file 12951_2023_2185_MOESM1_ESM.zip › figure S7.jpg]

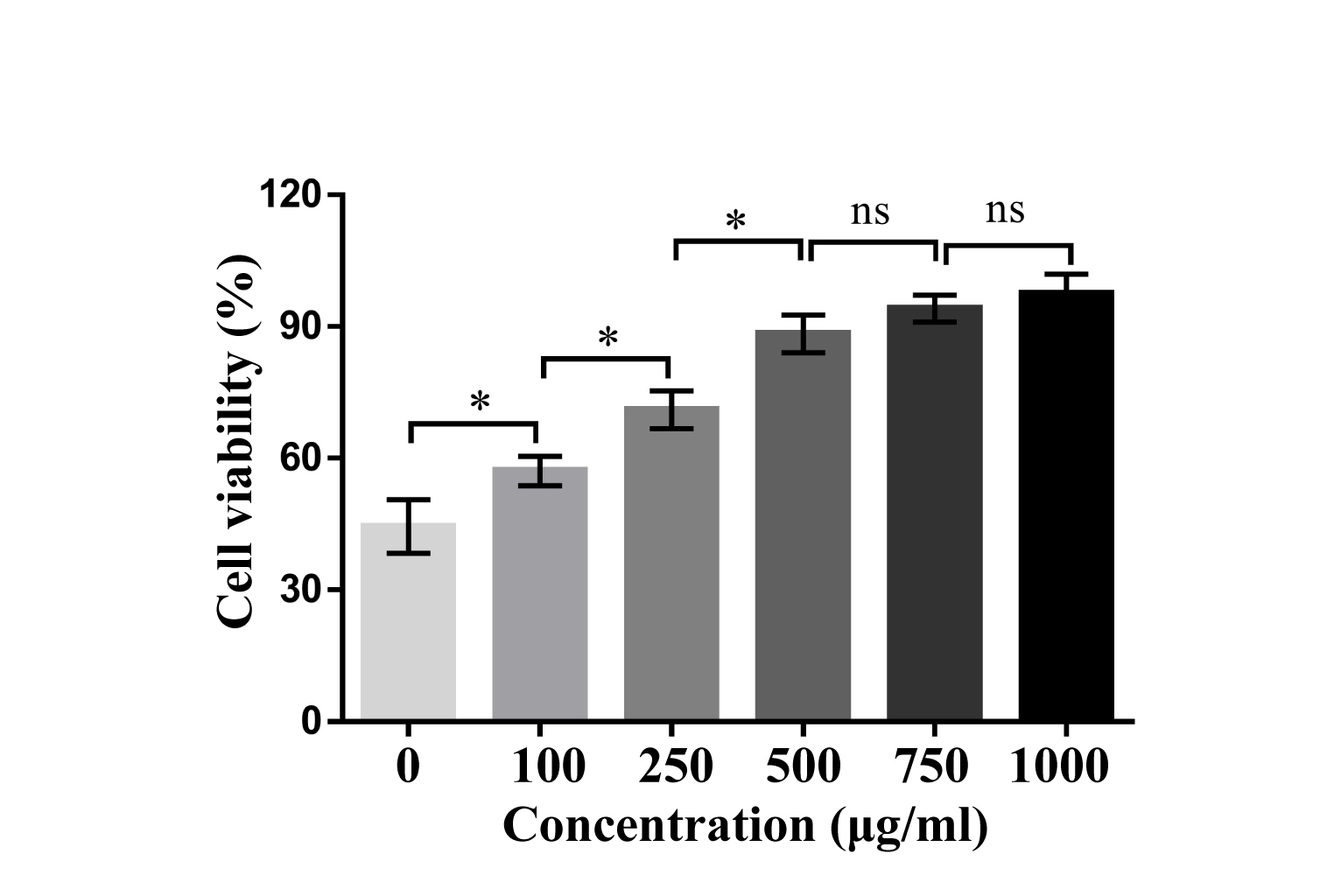

Supplement: Supplementary file 1 — Additional file 1: Figure S1. Identification of vascular endothelial cells (VECs). A Representative immunofluorescence staining of CD31 in VECs. B The proportion of CD31 + cells in extracted primary cells was determined by flow cytometry. Figure S2. Gene sequencing analysis of VECs treated with normoxia or hypoxia. A, B The volcano and Venn diagram of VECs treated with normoxia or hypoxia. C The differential expression of genes related to the ferroptosis and autophagy of VECs treated with normoxia or hypoxia was analyzed by heat map. D KEGG enrichment analysis of upregulated genes in hypoxic VECs compared with normoxic VECs. E GO enrichment analysis of upregulated genes in normoxic VECs compared with hypoxic VECs. Figure S3. Extracellular vesicles (EVs) inhibit the hypoxia-induced ferroptosis of VECs. A Propidium iodide (PI) staining of VECs treated with normoxia, hypoxia, EVs, or hypoxia + EVs was detected by flow cytometry. B, C The iron, MDA, and GSH levels and mitochondrial changes related to the ferroptosis of VECs treated as above. D, E Representative western blotting of GPX4 and mean fluorescence intensity (MFI) associated with reactive oxygen species (ROS) levels analyzed in VECs treated with normoxia, hypoxia, EVs, or hypoxia + EVs. ns: p > 0.05; *p < 0.05; **p < 0.01; ***p < 0.001. Figure S4. Ferroptosis was enhanced by autophagy in VECs. A KEGG enrichment analysis of all differential genes in VECs treated with or without hypoxia. B, C Representative western blot analysis of LC3A/B, ACSL4, GPX4, P53, P62, and lipid peroxidation in VECs treated with dimethyl sulfoxide (DMSO), erastin, or erastin + 3-methyladenine. D, E Iron, MDA, and GSH levels and mitochondrial changes associated with the ferroptosis of VECs treated as above. Figure S5. MFGE8 inhibited ferroptosis by diminishing autophagy in VECs. A Correlation analysis of MFGE8, ferroptosis-related proteins, and autophagy-related proteins. B–D Representative western blot analysis of P53, ACSL4, GPX4, LC3A [file 12951_2023_2185_MOESM1_ESM.zip › figure S8.jpg]

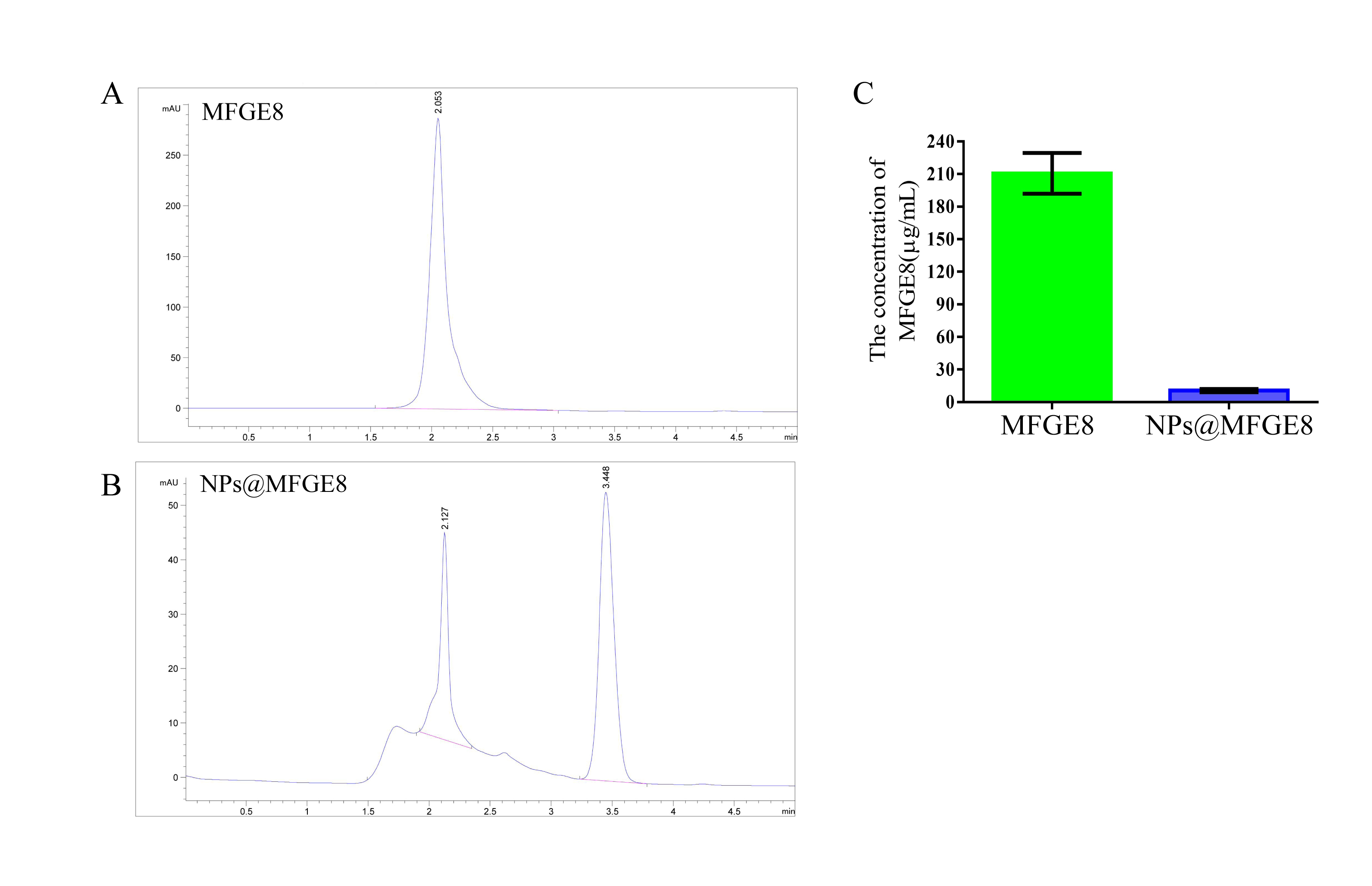

Supplement: Supplementary file 1 — Additional file 1: Figure S1. Identification of vascular endothelial cells (VECs). A Representative immunofluorescence staining of CD31 in VECs. B The proportion of CD31 + cells in extracted primary cells was determined by flow cytometry. Figure S2. Gene sequencing analysis of VECs treated with normoxia or hypoxia. A, B The volcano and Venn diagram of VECs treated with normoxia or hypoxia. C The differential expression of genes related to the ferroptosis and autophagy of VECs treated with normoxia or hypoxia was analyzed by heat map. D KEGG enrichment analysis of upregulated genes in hypoxic VECs compared with normoxic VECs. E GO enrichment analysis of upregulated genes in normoxic VECs compared with hypoxic VECs. Figure S3. Extracellular vesicles (EVs) inhibit the hypoxia-induced ferroptosis of VECs. A Propidium iodide (PI) staining of VECs treated with normoxia, hypoxia, EVs, or hypoxia + EVs was detected by flow cytometry. B, C The iron, MDA, and GSH levels and mitochondrial changes related to the ferroptosis of VECs treated as above. D, E Representative western blotting of GPX4 and mean fluorescence intensity (MFI) associated with reactive oxygen species (ROS) levels analyzed in VECs treated with normoxia, hypoxia, EVs, or hypoxia + EVs. ns: p > 0.05; *p < 0.05; **p < 0.01; ***p < 0.001. Figure S4. Ferroptosis was enhanced by autophagy in VECs. A KEGG enrichment analysis of all differential genes in VECs treated with or without hypoxia. B, C Representative western blot analysis of LC3A/B, ACSL4, GPX4, P53, P62, and lipid peroxidation in VECs treated with dimethyl sulfoxide (DMSO), erastin, or erastin + 3-methyladenine. D, E Iron, MDA, and GSH levels and mitochondrial changes associated with the ferroptosis of VECs treated as above. Figure S5. MFGE8 inhibited ferroptosis by diminishing autophagy in VECs. A Correlation analysis of MFGE8, ferroptosis-related proteins, and autophagy-related proteins. B–D Representative western blot analysis of P53, ACSL4, GPX4, LC3A [file 12951_2023_2185_MOESM1_ESM.zip › figure S9.jpg]

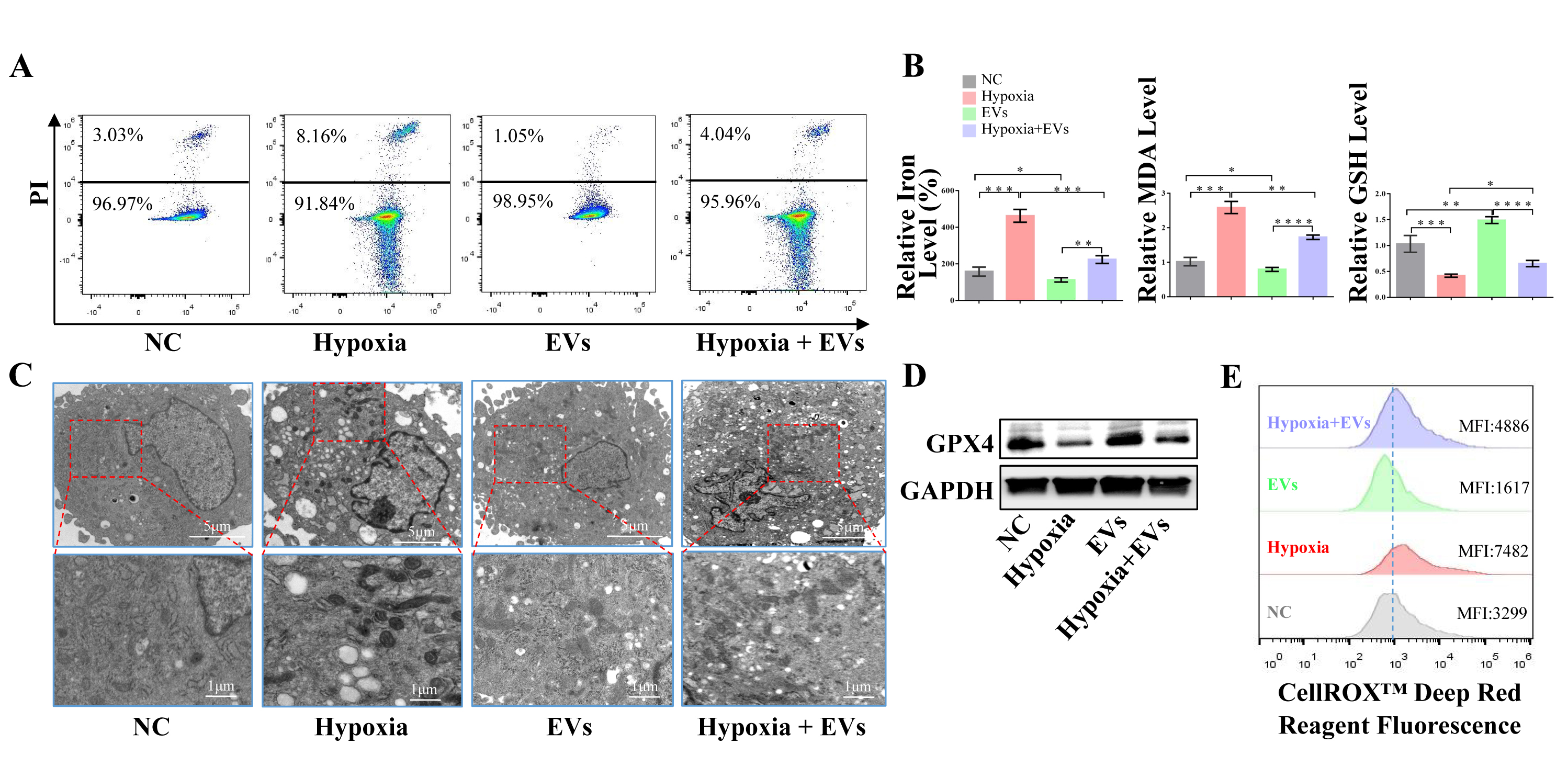

Supplement: Supplementary file 1 — Additional file 1: Figure S1. Identification of vascular endothelial cells (VECs). A Representative immunofluorescence staining of CD31 in VECs. B The proportion of CD31 + cells in extracted primary cells was determined by flow cytometry. Figure S2. Gene sequencing analysis of VECs treated with normoxia or hypoxia. A, B The volcano and Venn diagram of VECs treated with normoxia or hypoxia. C The differential expression of genes related to the ferroptosis and autophagy of VECs treated with normoxia or hypoxia was analyzed by heat map. D KEGG enrichment analysis of upregulated genes in hypoxic VECs compared with normoxic VECs. E GO enrichment analysis of upregulated genes in normoxic VECs compared with hypoxic VECs. Figure S3. Extracellular vesicles (EVs) inhibit the hypoxia-induced ferroptosis of VECs. A Propidium iodide (PI) staining of VECs treated with normoxia, hypoxia, EVs, or hypoxia + EVs was detected by flow cytometry. B, C The iron, MDA, and GSH levels and mitochondrial changes related to the ferroptosis of VECs treated as above. D, E Representative western blotting of GPX4 and mean fluorescence intensity (MFI) associated with reactive oxygen species (ROS) levels analyzed in VECs treated with normoxia, hypoxia, EVs, or hypoxia + EVs. ns: p > 0.05; *p < 0.05; **p < 0.01; ***p < 0.001. Figure S4. Ferroptosis was enhanced by autophagy in VECs. A KEGG enrichment analysis of all differential genes in VECs treated with or without hypoxia. B, C Representative western blot analysis of LC3A/B, ACSL4, GPX4, P53, P62, and lipid peroxidation in VECs treated with dimethyl sulfoxide (DMSO), erastin, or erastin + 3-methyladenine. D, E Iron, MDA, and GSH levels and mitochondrial changes associated with the ferroptosis of VECs treated as above. Figure S5. MFGE8 inhibited ferroptosis by diminishing autophagy in VECs. A Correlation analysis of MFGE8, ferroptosis-related proteins, and autophagy-related proteins. B–D Representative western blot analysis of P53, ACSL4, GPX4, LC3A [file 12951_2023_2185_MOESM1_ESM.zip › Fig. S3.jpg]

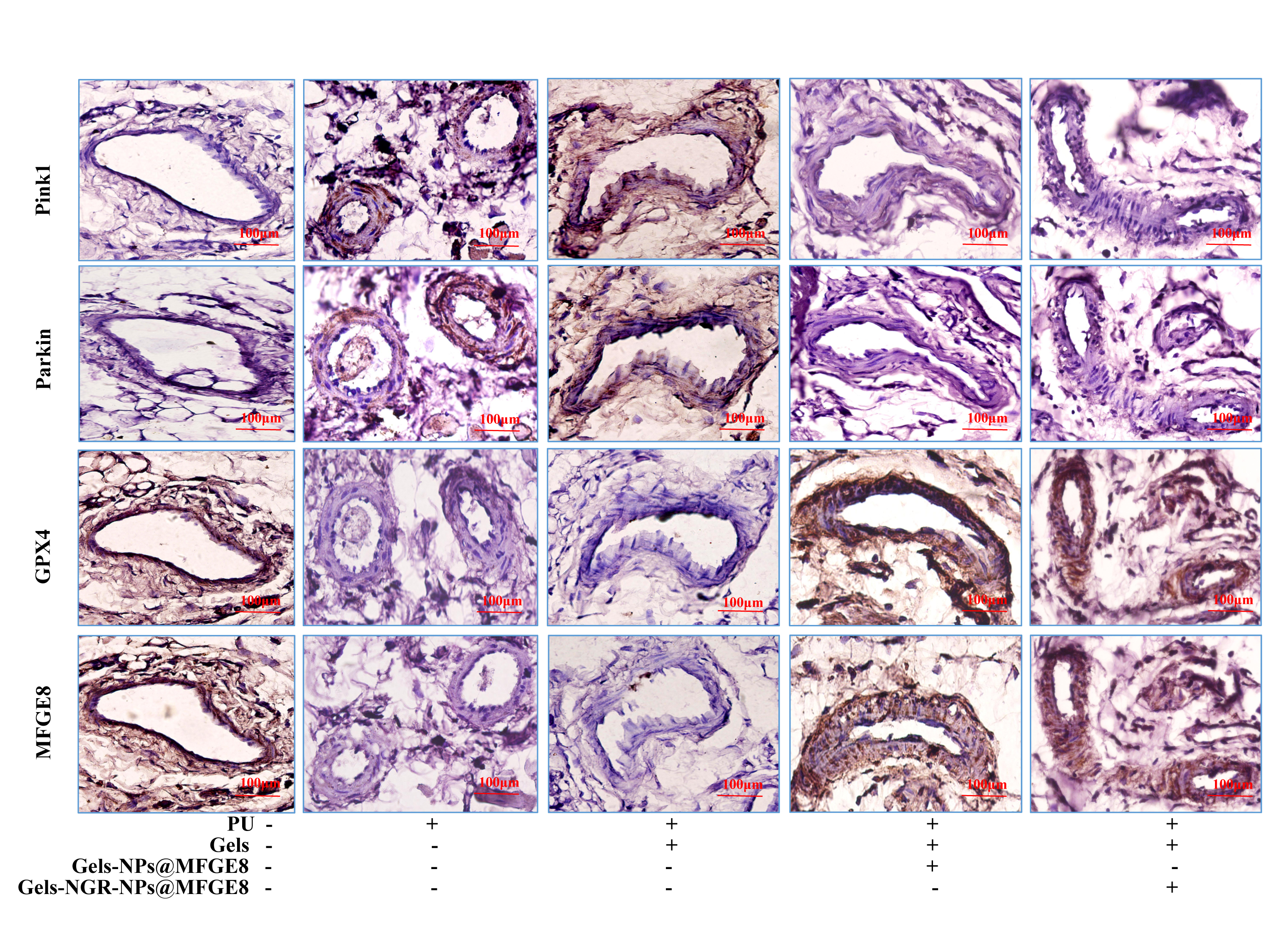

Supplement: Supplementary file 1 — Additional file 1: Figure S1. Identification of vascular endothelial cells (VECs). A Representative immunofluorescence staining of CD31 in VECs. B The proportion of CD31 + cells in extracted primary cells was determined by flow cytometry. Figure S2. Gene sequencing analysis of VECs treated with normoxia or hypoxia. A, B The volcano and Venn diagram of VECs treated with normoxia or hypoxia. C The differential expression of genes related to the ferroptosis and autophagy of VECs treated with normoxia or hypoxia was analyzed by heat map. D KEGG enrichment analysis of upregulated genes in hypoxic VECs compared with normoxic VECs. E GO enrichment analysis of upregulated genes in normoxic VECs compared with hypoxic VECs. Figure S3. Extracellular vesicles (EVs) inhibit the hypoxia-induced ferroptosis of VECs. A Propidium iodide (PI) staining of VECs treated with normoxia, hypoxia, EVs, or hypoxia + EVs was detected by flow cytometry. B, C The iron, MDA, and GSH levels and mitochondrial changes related to the ferroptosis of VECs treated as above. D, E Representative western blotting of GPX4 and mean fluorescence intensity (MFI) associated with reactive oxygen species (ROS) levels analyzed in VECs treated with normoxia, hypoxia, EVs, or hypoxia + EVs. ns: p > 0.05; *p < 0.05; **p < 0.01; ***p < 0.001. Figure S4. Ferroptosis was enhanced by autophagy in VECs. A KEGG enrichment analysis of all differential genes in VECs treated with or without hypoxia. B, C Representative western blot analysis of LC3A/B, ACSL4, GPX4, P53, P62, and lipid peroxidation in VECs treated with dimethyl sulfoxide (DMSO), erastin, or erastin + 3-methyladenine. D, E Iron, MDA, and GSH levels and mitochondrial changes associated with the ferroptosis of VECs treated as above. Figure S5. MFGE8 inhibited ferroptosis by diminishing autophagy in VECs. A Correlation analysis of MFGE8, ferroptosis-related proteins, and autophagy-related proteins. B–D Representative western blot analysis of P53, ACSL4, GPX4, LC3A [file 12951_2023_2185_MOESM1_ESM.zip › Fig. S12.jpg]
